# Cultivation-Based and Molecular Assessment of Bacterial Diversity in the Rhizosheath of Wheat under Different Crop Rotations

**DOI:** 10.1371/journal.pone.0130030

**Published:** 2015-06-29

**Authors:** Muhammad Tahir, M. Sajjad Mirza, Sohail Hameed, Mauricio R. Dimitrov, Hauke Smidt

**Affiliations:** 1 National Institute for Biotechnology and Genetic Engineering (NIBGE), Jhang Road Faisalabad, Punjab, Pakistan; 2 Laboratory of Microbiology, Wageningen University, Wageningen, The Netherlands; Agroecological Institute, CHINA

## Abstract

A field study was conducted to compare the formationand bacterial communities of rhizosheaths of wheat grown under wheat-cotton and wheat-rice rotation and to study the effects of bacterial inoculation on plant growth. Inoculation of *Azospirillum* sp. WS-1 and *Bacillus* sp. T-34 to wheat plants increased root length, root and shoot dry weight and dry weight of rhizosheathsoil when compared to non-inoculated control plants, and under both crop rotations. Comparing both crop rotations, root length, root and shoot dry weight and dry weight of soil attached with roots were higher under wheat-cotton rotation. Organic acids (citric acid, malic acid, acetic acid and oxalic acid) were detected in rhizosheaths from both rotations, with malic acid being most abundant with 24.8±2 and 21.3±1.5 μg g^-1^ dry soil in wheat-cotton and wheat-rice rotation, respectively. Two sugars (sucrose, glucose) were detected in wheat rhizosheath under both rotations, with highest concentrations of sucrose (4.08±0.5 μg g^-1^and 7.36±1.0 μg g^-1^) and glucose (3.12±0.5 μg g^-1^ and 3.01± μg g^-1^) being detected in rhizosheaths of non-inoculated control plants under both rotations. Diversity of rhizosheath-associated bacteria was evaluated by cultivation, as well as by 454-pyrosequencing of PCR-tagged 16S rRNA gene amplicons. A total of 14 and 12 bacterial isolates predominantly belonging to the genera *Arthrobacter*, *Azospirillum*, *Bacillus*, *Enterobacter* and *Pseudomonas*were obtained from the rhizosheath of wheat grown under wheat-cotton and wheat-rice rotation, respectively. Analysis of pyrosequencing data revealed *Proteobacteria*, *Bacteriodetes* and *Verrucomicrobia *as the most abundant phyla in wheat-rice rotation, whereas *Actinobacteria*, *Firmicutes*, *Chloroflexi*, *Acidobacteria*, *Planctomycetes* and *Cyanobacteria* were predominant in wheat-cotton rotation. From a total of 46,971 sequences, 10.9% showed ≥97% similarity with 16S rRNA genes of 32 genera previously shown to include isolates with plant growth promoting activity (nitrogen fixation, phosphate-solubilization, IAA production). Among these, the most predominant genera were *Arthrobacter*, *Azoarcus*, *Azospirillum*, *Bacillus*, *Cyanobacterium*, *Paenibacillus*, *Pseudomonas* and *Rhizobium*.

## Introduction

Crop rotation is a sequence of crops grown in a specific area over a specific period of time and is practiced to control pests, diseases and to maintain soil fertility by recharging the soil with nutrients. Crop rotations also have great influence on composition, richness and diversity of soil microbial communities [1], [2]. In Pakistan, wheat is grown mostly in two major types of rotations, i.e. wheat-cotton and wheat-rice crop rotation, whereas in South Asia, wheat-rice rotation is the major cereal production system [3]. During the growth period of rice, downward movement of various micronutrients takes place due to prolonged anoxic (reducing) conditions [3]. Consequently, deficiency of such elements is a common problem for cultivation of wheat when grown in rotation with rice [3]. To this end, a water management strategy that operates several dry-wet cycles alters the biological, chemical, and physical properties of soil under wheat-rice rotation. Consequently the nutrient status of the soil and its microbial community may change, which might in turn influence the growth of wheat cultivated in rotation with rice [4]. Cotton crop production requires high inputs of fertilizer like nitrogen, phosphorous and potassium (NPK). Incorporation of cotton stubble into the soil also increases the soil fertility, microbial diversity and growth of crop that follows cotton crop in rotation [5].

Soil microbial diversity and community structure depend on a number of factors like soil pH, temperature, moisture content, nature and amount of root exudates, crop rotations, soil nutrient status and agricultural practices [6]. Monoculture production results in low microbial diversity as compared to multicultural production [6], [7], and it has been observed that microbial community profiles in rhizosphere soil under continuous cultivation was different from those in soil under crop rotation [7].

The largest and most coherent rhizosheaths are formed around the roots of wheat and other grasses as the soil starts drying after irrigation, and they include soil particles, a network of root hairs and rhizosheath-associated microbial communities [8], [9]. Sheath formation requires fully hydrated exudates to permeate the surrounding soil particles that are then bonded to the root and each other as the mucilage dries. Rhizosheath soil was found to be significantly wetter than bulk soil, and it has been suggested that exudates within the rhizosheath increase the water holding capacity of the soil and nutrient acquisition [8], [9], [10]. Rhizosheaths act as an additional compartment in the rhizosphere of plants, being chemically and physically enriched and subsequently nourishing functional populations of microorganisms. Largely based on cultivation studies, rhizosheaths have been shown to harbour a range of different bacteria including members of the genera *Bacillus*, *Paenibacillus*, *Enterobacter* and *Agrobacterium* [11], [12].

Plant roots release carbon containing compounds including sugars, organic acids and amino acids into the rhizosphere as a result of rhizodeposition [13], [14], [15]. The composition of root exudates depends on the plant species, cultivar, plant growth stage, soil condition and plant growth substrate [15], [16]. Microorganisms present in soil, including beneficial bacteria, are attracted towards excreted compounds to utilize them as carbon and energy source, and secretion of root exudates was shown to increase the abundance, diversity and activity of rhizosphere bacteria [15], [16],[17].

Rhizosphere bacteria can improve plant growth by diverse mechanisms including phosphate solubilization, phytohormone production, nitrogen fixation and bio-control of plant pathogens [18], [19], [20],[21]. So-called plant growth promoting rhizobacteria (PGPR) are important for agriculture as a viable alternative to chemical fertilizers, and their application as biofertilizers can contribute to the reduction in the costs of crop production. A large number of PGPR, including isolates from the genera *Azospirillum*, *Azotobacter*, *Bacillus*, *Enterobacter*, *Pseudomonas*, *Klebsiella* and *Paenibacillus*, have been obtained from the rhizosphere of various crops [18], [19], [20], [21]. Application of PGPR as biofertilizer has resulted in improved growth and grain yield of various crops such as wheat, rice, maize and sugarcane [18], [19], [20],[21], [22].

The biosphere is dominated by microorganisms, yet only 0.1–10% microorganisms have been cultured [2], [23]. Therefore, microbial diversity cannot be studied comprehensively using cultivation-based approaches [24]. Culture-independent molecular approaches rely on extracted DNA for studying the composition and dynamics of microbial communities inhabiting soil as well as other environments. Such approaches have provided a new perspective in microbial ecology [2], [23], [24]. The information on microbial diversity and community structure of soil gathered through molecular studies is also important for agriculture as microorganisms are playing fundamental roles in biogeochemical cycles that drive plant health and nutrition [2].

The objective of this study was to investigate the formation of rhizosheaths, composition of root exudates with respect to organic acids and sugars, and the effect of bacterial inoculation with pre-selected PGPR on wheat growth under wheat-cotton and wheat-rice cropping rotations. Detailed information on the rhizosheath microbiology of wheat, especially under wheat-rice and wheat-cotton rotation is not available. Considering the extended anoxic and reducing conditions during rice cultivation as opposed to the generally oxic conditions in cotton field soils, we hypothesized the different crop rotations studied here would result in differences in the composition of microbial communities associated with wheat rhizosphere and rhizosheath. Therefore, we assessed bacterial community dynamics, using cultivation as well as 16S rRNA gene sequence based diversity analysis.

## Materials and Methods

### Field experimental design

A field experiment was conducted on experimental area of National Institute of Biotechnology and Genetic Engineering (NIBGE), Faisalabad to study the effect of crop rotation and PGPR inoculation on plant growth, rhizosheath formation, and the composition of bacterial communities associated with the rhizosheath of wheat grown under wheat-cotton and wheat-rice crop rotation. As no exotic bacterial strains or transgens were involved in the study, therefore specific permission from the Institute was not required for conduction of field experiments. Two previously isolated bacterial strains, *Bacillus* sp. T-34 and *Azospirillum* sp. WS-1, from the NIBGE Biotech Resource Centre (NBRC) were used as inoculants in this experiment along with a non-inoculated control. The experiment was laid out in Randomized Complete Block Design with three replicates and plot size of 6m × 4m. Bacterial cultures were grown overnight in 100 mL Lysogeny broth (LB medium; Trypton 1.0%, NaCl 0.5%, Yeast extract 0.5%, Agar 2%, pH 7±0.2) medium at 30°C on an arbitrary shaker at 150 rotations per min (rpm), and cells were harvested by centrifugation at 10,000 × g for 10 min. The cell pellet was washed with 0.85% saline solution and re-suspended in 100 mL of saline solution. The size of the inoculum was determined by serial dilution method. Ten-fold serial dilutions were made up to 10^−1^–10^-9^ in saline solution (0.85% NaCl), 100 μL aliquots from dilutions were spread on LB agar plates. The plates were incubated overnight at 30°C, and number of bacterial colonies (cfu) were counted and adjusted to 10^−9^ cfu.ml^-1^. The cell suspensions (100 mL) of bacterial strains were separately mixed with 250 g sterilized filter-mud (waste of sugarcane industry sterilized by autoclave at 121°C for 20 min). Seeds (0.4 kg) were then pelleted with one of the two mixtures containing filter mud and either strain T-34 or strain WS-1 at 10^9^ cfu.ml^-1^. Reduced levels of chemical urea and DAP (diammonium phosphate) fertilizer (equivalent to 80% of the dose recommended by Punjab Agriculture Department) was applied as nitrogen (0.31 kg. plot^-1^) and phosphorous (0.22 kg. plot^-1^) to each plot. Before sowing and fertilizer application, soil physico-chemical analysis was done. Under wheat-rice crop rotation, the soil was coarse loamy in nature with an electric conductivity (EC) of the saturation extract of 2.8 ds.m^-1^, pH 8.0, organic matter 0.58%, available phosphorous (P) 7.2 mg.kg^-1^ and total nitrogen (N) 0.057%. However, under wheat-cotton rotation the soil was coarse loamy, with an EC of 2.2 ds.m^-1^, pH 8.3, total N 0.061%, organic matter 0.62%, and available P 6.8 mg.kg^-1^. All other agronomic practices like irrigation, weed control and tillage were kept the same for all treatments throughout the growing season. To record the data on parameters including dry weight of soil attached with roots, root length, root and shoot dry weight,moisture contents of rhizosheath and bulk soil, three samples from each plot of a treatment were collected and pooled to get one composite sample. Similarly composite samples were collected from the remaining two plots of the same treatment, finally resulting in the collection of data of three replicates from each treatment. Data on dry weight of soil attached with roots, root length, root and shoot dry weight was recorded 35 days after sowing (DAS) and analyzed. All further analyses were done on soil samples taken at 6–7% moisture content of bulk soil when rhizosheaths were formed around the roots at 30–35 DAS. Moisture content (Gravimetric water content %) of bulk soil and rhizosheaths was determined using the formula, Soil Water Content (SWC) = % water (w/w) = [(Wl−W2)/W2] × 100, [9], where W1 is the fresh weight of soil, and W2 is the dry weight of soil after drying the soil at 105°C for 5 days.

When measuring root length, roots of each plant were removed separately, washed in water and spread on a transparent polyethylene sheet. The sheet with roots was scanned using a desktop scanner (CanoScan, Canon, Lide 110, Japan) and root length was measured using a root image analysis program (Washington State University Research Foundation program, Washington State University, USA).

### Estimation of phosphorous and nitrogen contents in plant materials

Plant material was oven dried and ground in a stainless steel grinding mill. For estimation of nitrogen and phosphorus, wet ashing of oven dried plant material was carried out with H_2_SO_4_ and H_2_O_2_ and processed as described by Van Schouwenberg and Walinge (1973) and Murphy and Riley (1962) [25], [26].

### Extraction and analysis of organic acids and sugars in rhizosheaths

Rhizosheath soil samples were collected from wheat growing under wheat-rice and wheat-cotton rotations at tillering stage. To measure the weight of rhizosheath soil, roots with attached soil were washed in pre-weighed Falcon tubes containing 30 mL sterilized distilled water for 5–10 min. The weight of rhizosheath soil was defined as the difference in weight of tubes before and after washing. The suspended soil was centrifuged at 6000 × g for 8 min. Supernatant was collected and concentrated to 1.5 mL in a concentrator (Concentrator 5301, Eppendorf, Germany) and filtered over a 0.2 μm filter (Orange Scientific GyroDisc CA-PC, Belgium). Filtrates were analyzed by HPLC, using a PERKIN ELMER series 200 with 20 μL auto-sampler PE NELSON 900 series interface, PE NELSON 600 series link and PERKIN ELMER NCI 900 Network Chromatography interface using Diode-array detector at 210 nm and their UV spectra (190–400 nm), Microgaurd Cation-H Precolumn and an Aminex HPx-87H analytical column for separation. Sulfuric acid (0.001N) was used as mobile phase with flow rate of 0.6 mL min^-1^. Solutions (100 μg.L^-1^) of citric acid, malic acid, acetic acid, succinic acid, gluconic acid, lactic acid and oxalic acid were used as standards. For determination of sugars the same eluent and flow rate as for organic acids were maintained. Detection was made on refractive index detector (Shimadzu Co., Japan) by keeping the column temperature at 85°C. The solutions (100 μg.L^-^1) of sugars (sucrose, maltose, glucose, galactose and xylose) were used as standards. Peak area and retention time of samples were compared with those of standards for quantification of organic acids and sugars.

### Cultivation, isolation and identification of bacteria from rhizosheath and bulk soil

The composition of the bacterial communities associated with rhizosheaths and bulk soil was assessed at 35 DAS. Ten-fold serial dilutions were made up to 10^−3^–10^−5^ in saline solution (0.85% NaCl), and 100 μL aliquots from dilutions were spread on LB agar plates [27] which is an enriched growth medium suitable for the isolation and growth most of soil bacteria [22]. The plates were incubated for 12–72 hours at 30°C, and morphologically different colonies were selected for further purification and identification. Isolated colonies were re-streaked on fresh LB agar plates and incubated at 30°C to obtain single colonies. For isolation of diazotrophic bacteria, semi-solid nitrogen-free medium (NFM) [28] was inoculated with aliquots (100 μL) of 10-fold serial dilutions of soil. Enrichment cultures were obtained by repeated transfers in fresh NFM medium after every transfer at 30°C for 72 hours. After five consective transfers, 100 μL of bacterial growth in NFM medium were spread on agar plates of NFM and LB media to get single bacterial colonies. Bacterial isolates were identified using 16S rRNA gene sequence analysis. Bacteria were grown overnight in LB broth at 30°C, bacterial cell pellets were obtained by centrifugation at 10,000 × g, and genomic DNA was extracted using the FastDNA SPIN Kit for Soil (MP Biomedicals, USA) following the manufacturer’s instructions. DNA concentration and purity was measured using a NanoDrop® ND-1000 spectrophotometer (NanoDrop Technologies, Inc- USA). The 16S rRNA gene of the isolates was amplified by adding 1.0 μL (40 ng) of DNA to a PCR mix, containing 10 μL of 5× buffer, 1.0 μL of dNTPs (final concentration 200 μM), 0.2 μL of each primer at 10 μM (forward and reverse), 0.5 μL (5U μL^-1^) of Phire®enzyme (Thermo Scientific, USA) and nuclease-free water up to 50 μL. Primers used for 16S rRNA gene amplification were 27F: 5’-GGGGTTTGATCCTGGCTCAG-3’ and 1492R: 5’-CGGCTACCTTGTTACGAC-3’ [29]. The following PCR cycling conditions were used: Denaturation at 98°C for 2 min, followed by 35 cycles of 98°C for 10 sec, 52°C for 10 sec and 72°C for 30 sec, and a final extension step at 72°C for 10 min. PCR products were checked for correct size by agarose gel electrophoresis, purified using the High Pure PCR Cleanup Micro Kit (Roche Applied Science, Germany). After purification, concentration of PCR products was measured on NanoDrop. Reaction mixture was made by adding 2.5 μL of 27 F primer (10 μM), PCR product and water to make the volume of mixture up to 20 μL. Samples were sent for sequencing on capillary sequencers (Baseclear, Leiden, The Netherlands). The 16S rRNA gene sequences obtained were trimmed (Bio-Edit 7.1), compared with publicly available sequences using BLAST [30] at the National Centre for Biotechnology Information web site (www.ncbi.nlm.nhi.gov), and submitted to EMBL.

### Extraction of DNA from rhizosheaths

Rhizosheath samples were taken at 35 DAS from inoculated and non-inoculated wheat plants at 6–7% moisture level and were stored at 4°C until further processing. Soil samples were collected (five replicates) from each inoculated and non-inoculated plot and mixed to get one pooled sample. From each pooled sample, two independent soil DNA extractions were made and each DNA extract was subjected to PCR reactions. Similarly another independent soil sample was collected from a different replicate of the same treatment and processed in the same way as discussed above. As a result total 12 soil samples (two from each treatment) were processed for further analysis. Soil DNA was extracted and quantified as described above for pure bacterial cultures.

### Amplification of 16S rRNA gene fragments for next generation sequencing

Extracted rhizosheath DNA was diluted to 20 ng.μL^-1^. Amplicons from the V1 to V2 region of 16S rRNA genes were generated by PCR using forward primer 27F and an equimolar mix of reverse primers 338R-I+II (Table 1). To allow pyrosequencing using titanium chemistry, each forward primer was appended with the titanium sequencing adaptor A and an “NNNN” barcode sequence (Table 1) at the 5’-end, where NNNN is a sequence of eight nucleotides that was unique for each sample and did neither start with G nor contained a homotrimer of any of the four bases. The reverse primer carried the titanium adaptor B at the 5’-end. PCR mix was prepared using 20.0 μL 5× HF buffer, 5.0 μL of an equimolar mix of reverse primers TitAdapB338R-I and TitAdapB338R-II (10 μM of each primer) [31], [32], [33], 2.0 μL of dNTP (final concentration 200 μM), 1.0 μL Phusion Hot start II DNA polymerase (2U μL^-1^), 65.0 μL nuclease free water to make 93.0 μL total volume for each sample. For amplification, 5.0 μL barcoded forward primer 27F and 2.0 μL (20 ng.μL^-1^) template DNA were added to 93.0 μL PCR mix. The PCR cycle conditions were the same as described previously [33] with some modifications. Initial denaturation was done at 98°C for 30 sec, followed by 30 cycles of 98°C for 10 sec, 56°C for 20 sec and 72°C for 20 sec, and a final extension at 72°C for 10 min. PCR products were analyzed by agarose gel electrophoresis, and purified with High Pure PCR Cleanup Micro Kit (Roche Applied Science, Germany) using manufacturer’s instructions. DNA concentration after purification was measured using a NanoDrop® ND-1000. All purified PCR products (two replicates of each treatment i.e. a total of 12 amplicons) were mixed in equimolar amounts with a final DNA concentration of 100 ng.μL^-1^. Pooled amplicons were analyzed on agarose gel, the corresponding band was excised, DNA was eluted using DNA Gel Extraction Kit (Millipore) and quantified as described above. Pyrosequencing was performed on half a picotiter plate using a FLX genome sequencer in combination with titanium chemistry (GATC-Biotech, Konstanz, Germany).

**Table 1:** Adaptors and primers used for targeting bacterial communities present in the rhizosheath of wheat grown under different crop rotations.

Rotation	Sample ID	Barcode sequence	Primers (5’- 3’)
**wheat-cotton rotation**	A1 Soil *Bacillus* T-34 R1	AACGCGAA	27F-DegS, GTTYGATYMTGGCTCAG, [33]; 338R-I, GCWGCCTCCCGTAGGAGT, [31]; 338R-II, GCWGCCACCCGTAGGTGT, [31]
	A3 Soil *Bacillus* T-34 R3	AACGCGTT	
	B1 Soil *Azospirillum* WS-1 R1	AAGCGCTT	
	B3 Soil *Azospirillum* WS-1 R3	ACACCTGA	
	E1 soil non-inoculated R1	AAGCGGAT	
	E3 soil non-inoculated R3	AAGCGGTA	
**wheat-rice rotation**	1.1 Soil *Bacillus* T-34 R1	AAGCTACC	
	1.3 Soil *Bacillus* T-34 R3	AAGCTAGG	
	2.1 Soil *Azospirillum* WS-1 R1	AAGCTTCG	
	2.3 Soil *Azospirillum* WS-1 R3	AAGCTTGC	
	5.1 soil non-inoculated R1	ACACCTCT	
	5.3 soil non-inoculated R3	ACACGACT	

### Sequence data analysis

Sequences were processed and sorted using the default parameters in QIIME version 1.3 [34]. Briefly, sequences were submitted to a quality check, and only high-quality sequences (>200 bp and <1000 bp in length, quality score >25, exact match to barcode and primer) were selected for further processing. High-quality sequences were clustered into operational taxonomic units (OTUs) at 97% sequence identity using UCLUST. Representative sequences of each OTU were then aligned using PyNAST [35] against the Greengenes core set [36], and taxonomy was assigned using the RDP-classifier [37]. Chimeric sequences were removed using ChimeraSlayer software [38], and aligned non-chimeric sequences were used to generate a phylogenetic tree using FastTree [39]. In order to eliminate potential biases introduced by sampling depth, all samples were rarified to an equal number of sequences per sample. Association of microbial communities with different treatment applied in this study was assessed using Principle Coordinate Analysis (PCoA) of weighted UniFrac distances as implemented in QIIME.Sequence data obtained from pyrosequencing were submitted to the NCBI Sequence Read Archive (SRA) under accession number SRP045888."

### Phylogenetic analysis

Closely related sequences were downloaded and aligned using CLUSTAL X. These sequences were analyzed using neighbor joining method. The bootstrap replicates (BS) values of 50% or greater represent well supported nodes and thus only those were retained.

### Statistical analysis

Results obtained from plant growth parameters were subjected to analysis of variance (ANOVA), and significance at the 5% level was tested by Least Significance Difference Test (LSDT) by using software STATISTIX (8.1 version). Mean values, coefficient of variation and the standard deviations were calculated.

## Results

**Fig 1:**
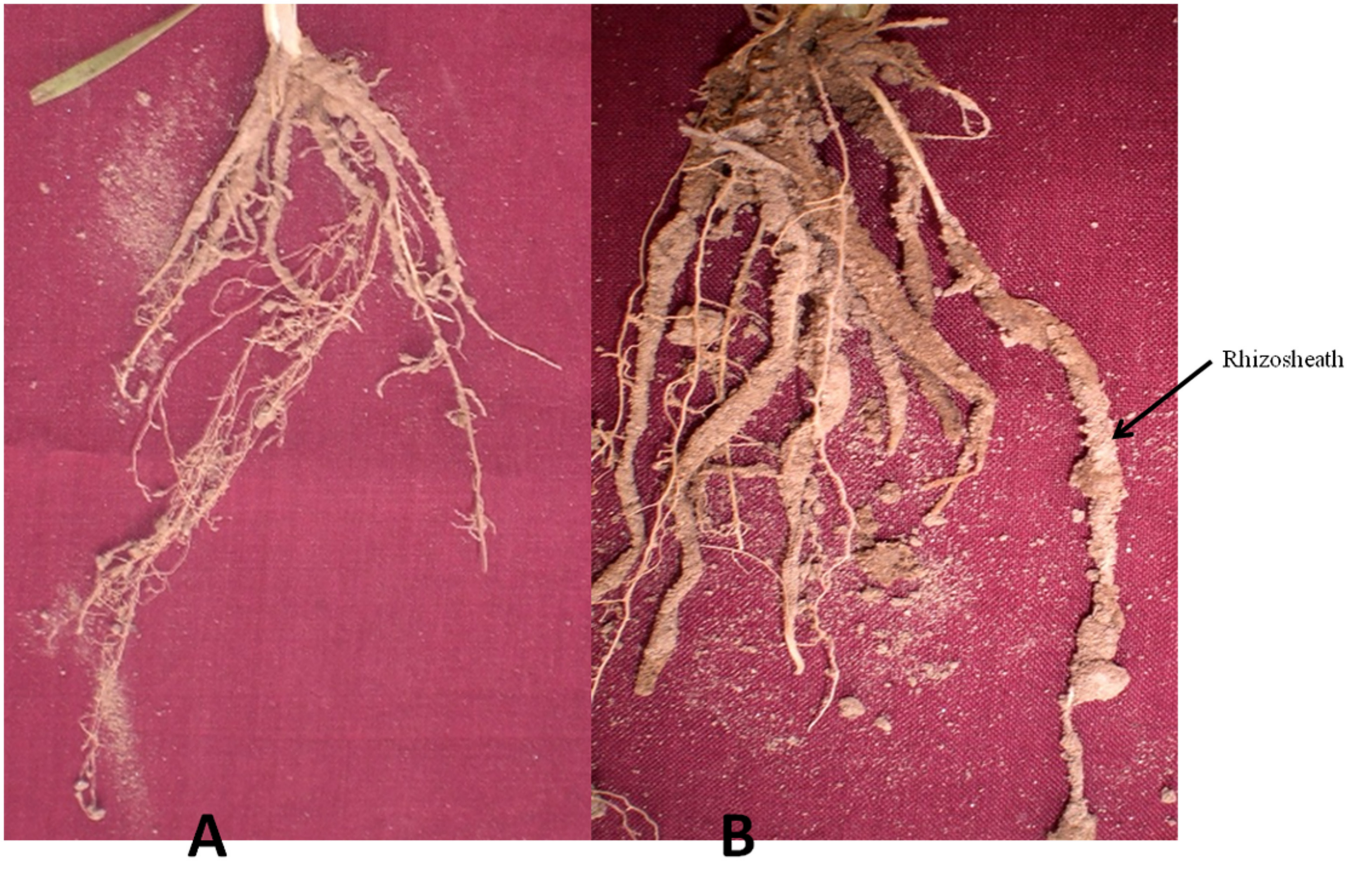
Representative images of rhizosheath formation in wheat grown in wheat-rice (A) or wheat-cotton (B) rotation. Plants were uprooted along with root system carefully. Soil directly attached with roots was extracted and used for further studies.

In the present study, we set out to assess the effect of different wheat-containing crop rotation systems as well as inoculation with previously isolated plant growth promoting rhizosphere bacteria on plant growth and physical-chemical and microbial characteristics of wheat-associated rhizosheaths. To this end, rhizosheath formation was observed around the roots of wheat under both crop rotations, albeit much stronger around the inoculated roots under wheat-cotton rotation compared to that of inoculated roots under wheat-rice rotation (Fig 1A and 1B). Soil moisture content was determined 35 DAS in the bulk soil and rhizosheath of wheat. In the bulk soil of wheat-cotton rotation moisture content was 6.8±0.3% (average of three treatments) where as the corresponding moisture content of rhizosheaths was 11.5±1.1%. Moisture content of bulk soil and rhizosheath under wheat-rice rotation was 7.3±0.4% and 9.3±0.6% (average of three treatments), respectively (Table 2). Inoculation of *Azospirillum* strain WS-1 increased the moisture contents by 18.2% and 8.8% under wheat-cotton and wheat-rice rotation respectively over non-inoculated control. Moisture contents of rhizosheath and bulk soil did not significantly differ between the three treatments. However, moisture contents determined in rhizosheath of plants inoculated with *Azospirillum* sp. WS-1 or *Bacillus* sp. T-34 were always higher than in rhizosheath of non-inoculated plants under both crop rotations (Table 2).

**Table 2:** Moisture contents (%) determined in rhizosheath and bulk soil of wheat grown under wheat-rice and wheat-cotton rotation.

Treatments	Moisture contents of rhizosheath (%) under wheat-cotton rotation	Moisture contents of rhizosheath (%) under wheat-rice rotation
*Azospirillum* sp. WS-1	12.3±1.0	9.8±0.5
*Bacillus* sp. T-34	12.0±0.8	9.2±0.5
Non-inoculated	10.4±1.5	9.0±0.7
**Average of both rotations**	**11.5±1.1**	**9.3±0.6**
**Treatments**	Moisture contents of bulk soil (%) under wheat-cotton rotation	Moisture contents of bulk soil (%) under wheat-rice rotation
***Azospirillum* sp. WS-1**	7.0±0.2	7.6±0.4
***Bacillus* sp. T-34**	6.8±0.4	7.3±0.3
**Non-inoculated**	6.6±0.4	7.0±0.5
**Average of both rotations**	**6.8±0.3**	**7.3±0.4**

Moisture contents (%) in rhizosheath were determined by collecting the soil attached with roots as rhizosheathand bulk soil from each treatment in three replicates using the formula;

Soil Water Content (SWC) = % water (w/w) = [(Wl−W2)/W2] × 100 [9], where W1 is the fresh weight of soil, and W2 is the dry weight of soil after drying the soil at 105°C for 5 days

Rhizosheath soil was separated from the roots and its dry weight was recorded. A higher dry weight of soil in the rhizosheath of wheat was noted in the plants grown under wheat-cotton as compared to wheat-rice rotation (1.09±0.05 g vs. 0.21±0.02 g; mean of three treatments in each rotation) (Table 3). Furthermore, it was observed that soil attachment was influenced by inoculation of bacterial strains under both crop rotations. Dry weight of rhizosheaths was greater (*P*<0.05) in plants inoculated with *Bacillus* sp. T-34 and *Azospirillum* sp. WS-1 (1.34±0.05 and 1.32±0.04 g, respectively) compared to non-inoculated control plants under wheat-cotton rotation (0.62±0.03 g) (Table 3). Similarly, under wheat-rice rotation, higher (*P*<0.05) dry weight of rhizosheath soil was found for *Bacillus* and *Azospirillum* inoculated plants when compared with non-inoculated plants (0.27±0.02 and 0.23±0.01 g vs. 0.13±0.02 g; Table 3).

**Table 3:** Effect of bacterial inoculation on rhizosheath formation and growth of wheat under different crop rotations.

Crop rotation	Inoculum	Dry weight of soil attached with roots as rhizosheath r(g/plant)	Root length (cm)	Shoot dry weight (g)	Root dry weight (g)
**Wheat-cotton rotation**	***Bacillus* sp. T-34**	1.34 A	119.8A	0.6700A	0.21 A
	***Azospirillum* sp. WS-1**	1.32 A	110.5B	0.4333B	0.18 B
	**Control**	0.62 B	104.9B	0.3567C	0.17 B
	**Mean**	**1.09 A**	**111.7A**	**0.4867A**	**0.19 A**
**Wheat-rice rotation**	***Bacillus* sp. T-34**	0.27 A	92.5 A	0.2033A	0.17 A
	***Azospirillum* sp. WS-1**	0.23 A	81.6 B	0.1900B	0.17 A
	**Control**	0.13 B	48.5 C	0.1800B	0.09 B
	**Mean**	**0.21 B**	**74.2 B**	**0.1911B**	**0.14 B**

Plants were grown in field in randomized complete block design with three replicates and uprooted along with root system 35 DAS. The values are an average of three replicates.

Higher (*P*<0.05) cumulative root length (111.7±5 cm/plant, mean value of three treatments, Table 3) was observed in the plants grown under wheat-cotton rotation compared to those from wheat-rice rotation (74.2±6.5 cm/plant). With respect to the bacterial treatments, inoculation with *Bacillus* sp. strain T-34 produced longer roots (119.8±5 and 92.5±4.5 cm/plant, respectively, under wheat-cotton and wheat-rice rotation) compared to non-inoculated control (104.9±4 and 48.5±4 cm/plant). Root dry weight (0.19±0.01 g, mean value of three treatments) and shoot dry weight (0.4867±0.02 g, mean value of three treatments) were higher (*P*<0.05) in plants taken from wheat-cotton rotation when compared to root dry weight (0.14±0.01 g) and shoot dry weight (0.1911±0.02 g) of plants taken from wheat-rice rotation. Furthermore the inoculation of *Azospirillum* sp. WS-1 also increased (*P*<0.05) the root and shoot dry weight over non-inoculated control plants. Root length and root dry weight was increased by 14.4% and 23.5% respectively, due to inoculation of *Bacillus* strain T-34 over non-inoculated control plants under wheat-cotton rotation (Table 3). Nitrogen (mg/g of dry plant material) and phosphorous contents (%) were determined. Data showed that inoculation of *Azospirillum* sp. WS-1 slightly increased the nitrogen contents under wheat-rice and wheat-cotton rotation (0.90 mg.g^-1^ of dry plant material and 0.98 mg.g^-1^ respectively) over non-inoculated control plants (0.80 mg.g^-1^ and 0.74 mg.g^-1^ respectively under wheat-rice and wheat-cotton rotation). Phosphorous contents were slightly higher in the dry material of the plants inoculated with *Bacillus* sp. T-34 under wheat-rice and wheat-cotton rotation (0.43% and 0.48% respectively) as compared to non-inoculated control plants (0.40% and 0.43% respectively).

**Fig 2:**
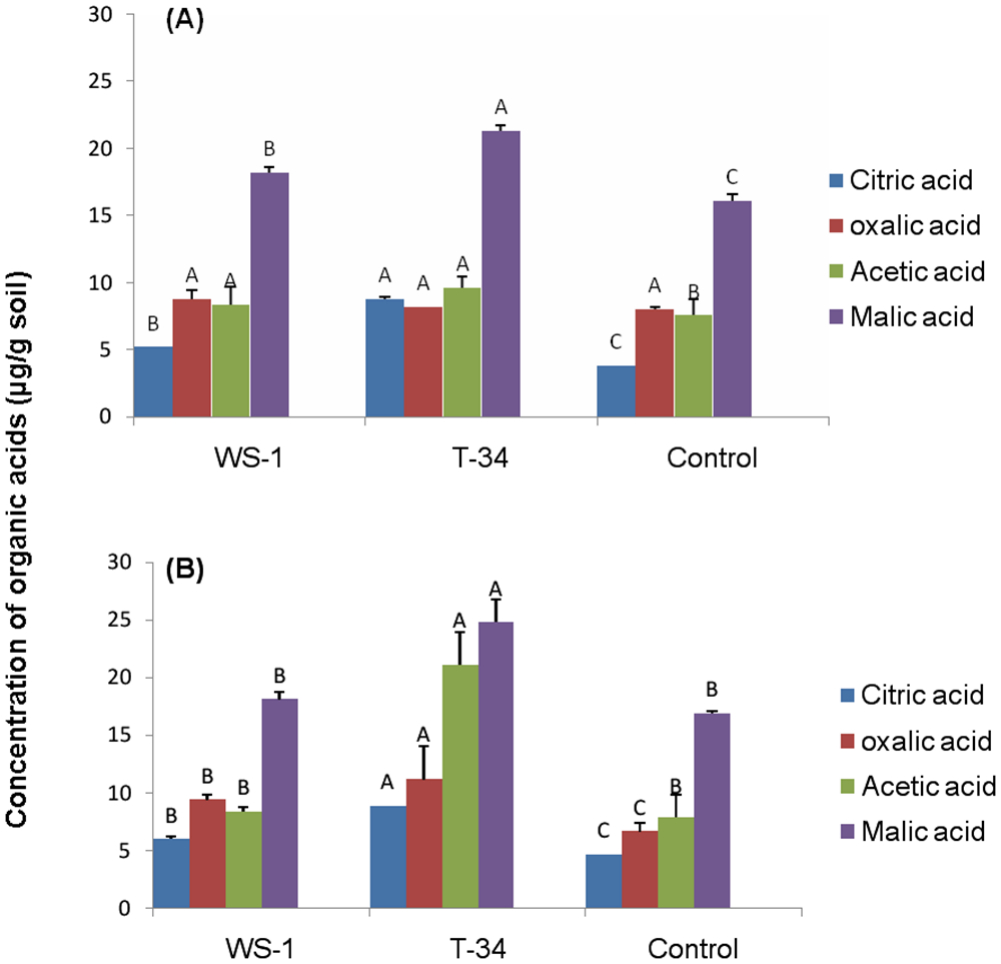
Detection of organic acids (μg.g^-1^ dry soil) in rhizosheaths of wheat. **Wheat-cotton (B) and wheat-rice (A) crop rotations.** Plants were inoculated with *Azospirillum* sp. WS-1, *Bacillus* sp. T-34 or non-inoculated (control). Organic acids were collected by washing the roots containing soil as rhizosheath in 30 mL sterilized distilled water for 5–10 minutes. The values given are an average of 3 replicates. Letters A, B and C represent the significance level by comparison of means.

HPLC analysis of organic acids extracted from the rhizosheaths showed that citric acid, oxalic acid, acetic acid and malic acid were detected in rhizosheaths of wheat under both crop rotations. Among the treatments, *Bacillus* sp. T-34 inoculated plant roots under the both crop rotations secreted higher amounts of organic acids (Fig 2A and 2B). In this treatment higher amounts of citric acid (8.86±0.11 and 8.81±0.04 μg.g^-1^ of dry soil, under wheat-cotton rotation and wheat-rice rotation, respectively) were observed as compared to that detected in rhizosheaths of non-inoculated plants grown under wheat-cotton rotation (3.79±0.03 μg.g^-1^ of dry soil) and wheat-rice crop rotation (4.62±0.06 μg.g^-1^ of dry soil). In the same treatment malic acid concentrations were significantly higher in the rhizosheath (24.79±0.43 μg.g^-1^ of dry soil under wheat-cotton rotation and 21.34±1.97 μg.g^-1^ of dry soil under wheat-rice rotation) when compared with that detected in rhizosheath of non-inoculated control plants (16.07±0.53 μg.g^-1^ under wheat-cotton rotation and 16.9±0.18 μg.g^-1^ dry soil under wheat-rice rotation). Oxalic acid concentration was significantly (*P*<0.05) higher (11.13±2.9 μg.g^-1^ of dry soil) in rhizosheath of plants inoculated with *Bacillus* sp. T-34 under wheat-cotton rotation when compared to that detected in the rhizosheath of non-inoculated plants (6.65±0.73 μg.g^-1^ of dry soil) under this system (Fig 2B). In contrast, the concentration of this acid in the rhizosheath was not significantly (*P*<0.05) affected by bacterial inoculation under wheat-rice rotation (Fig 2A). Acetic acid concentration was significantly (*P*<0.05) higher (21.1±2.8 μg.g^-1^ of dry soil) in the rhizosheath inoculated with *Bacillus* sp. T-34 when compared with that detected in rhizosheath of plants inoculated with *Azospirillum* sp. WS-1 (8.35±1.9 μg.g^-1^ of dry soil) and non-inoculated control plants (7.88±0.4 μg g^-1^ of dry soil) under wheat-cotton rotation (Fig 2B). Similarly higher concentrations of acetic acid (9.63±0.9 μg.g^-1^ of dry soil) were detected in the rhizosheath of plants inoculated with *Bacillus* sp. T-34 when compared with that observed in rhizosheath of plants inoculated with *Azospirillum* sp. WS-1 (8.37±1.2 μg.g^-1^ of dry soil) and non-inoculated rhizosheath (7.56±1.3 μg.g^-1^ of dry soil) under wheat-rice rotation (Fig 2A).

**Fig 3:**
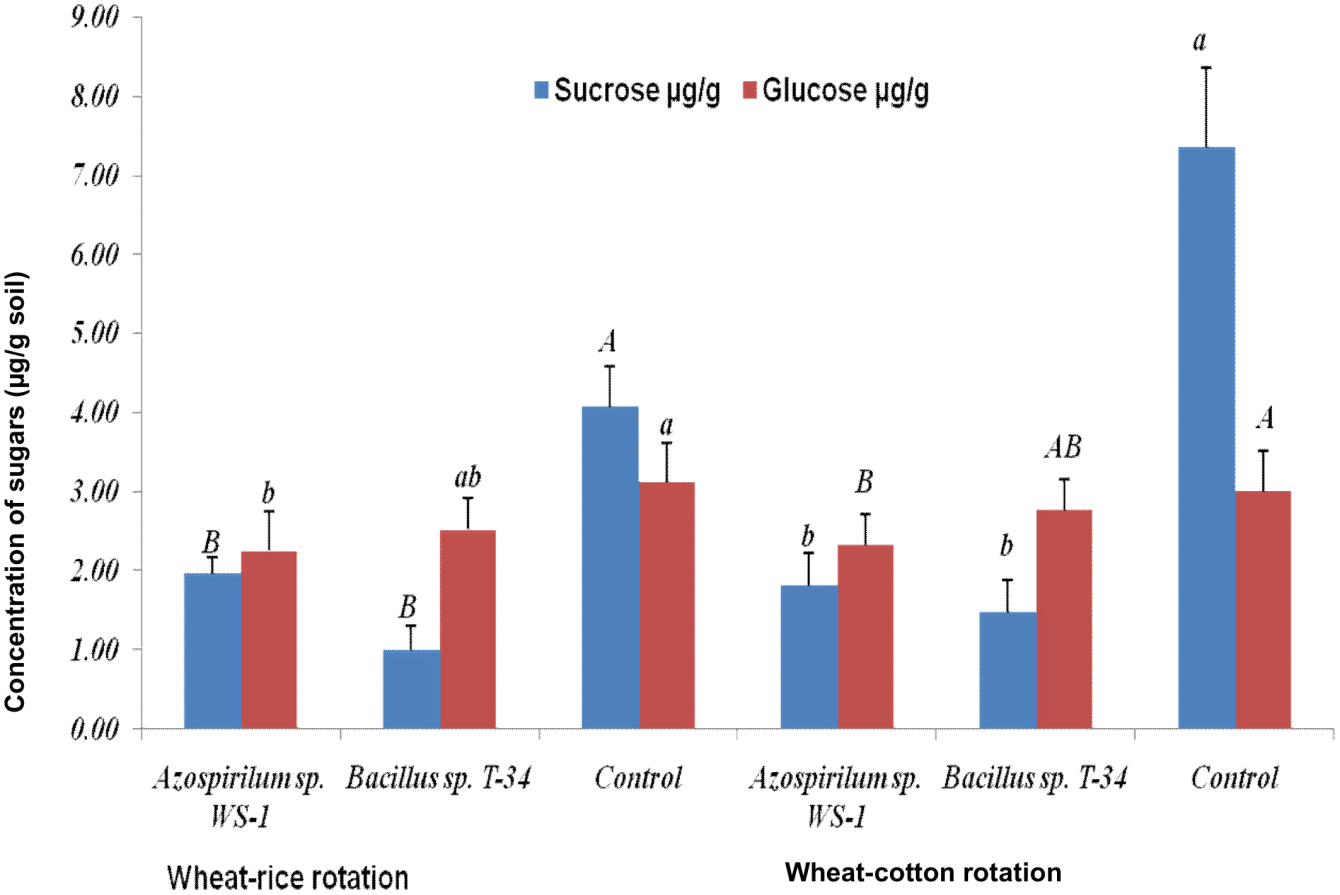
Detection of sugars (μg.g^-1^ dry soil) in rhizosheaths of wheat. Plants were inoculated with *Azospirillum* sp. WS-1, *Bacillus* sp. T-34 or non-inoculated (control). Sugars were collected by washing the roots containing soil as rhizosheath in 30 mL sterilized distilled water for 5–10 minutes. The values given are an average of 3 replicates. Letters Aa, Bb and Cc represents the significance level by comparison of means.

In the present study two sugars were detected in the rhizosheath of wheat grown under either crop system. Under wheat-rice rotation sucrose concentration was 2.35±0.41 μg.g^-1^ dry soil, whereas glucose was 2.63±0.3 μg.g^-1^ dry soil (Fig 3). Higher amounts were detected under wheat-cotton rotation, where sucrose concentration was 3.55±0.5 μg.g^-1^ of dry soil and glucose was 2.69±0.3 μg.g^-1^ of dry soil (Fig 3). However, comparing treatments, higher (*P*<0.05) sucrose (4.08±0.5 μg.g^-1^ and 7.36±1.0 μg.g^-1^, wheat-rice and wheat-cotton, respectively) and glucose concentrations (3.12±0.5 μg.g^-1^ and 3.01± μg.g^-1^, wheat-rice and wheat-cotton, respectively) were detected in the rhizosheath of non-inoculated control plants (Fig 3).

**Fig 4:**
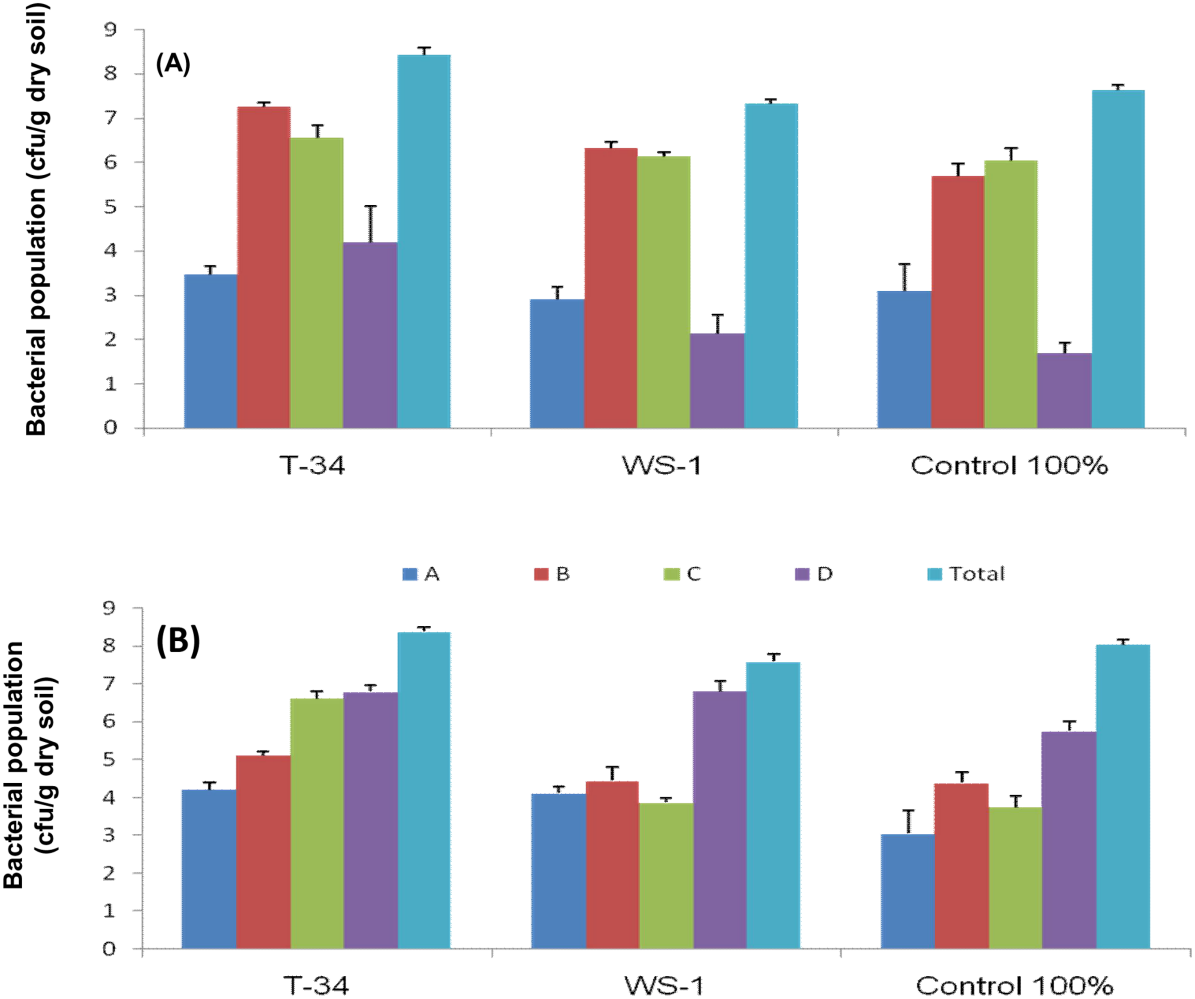
Population of culturable bacteria (cfu.g^-1^ of soil) on LB medium in rhizosheath of wheat. Wheat-rice rotation (A) and wheat-cotton rotation (B). Plants were inoculated with *Azospirillum* sp. WS-1, *Bacillus* sp. T-34 or non-inoculated (control). Bacterial population sizes were measured in rhizosheath soil samples by plating of serial dilutions on LB agar. Log values were calculated for colony forming units (cfu). Given values are the mean of 3 replications. Four different predominant types of colonies (A, B, C, D) could be distinguished as based on 16S rRNA gene sequence analysis.

**Fig 5:**
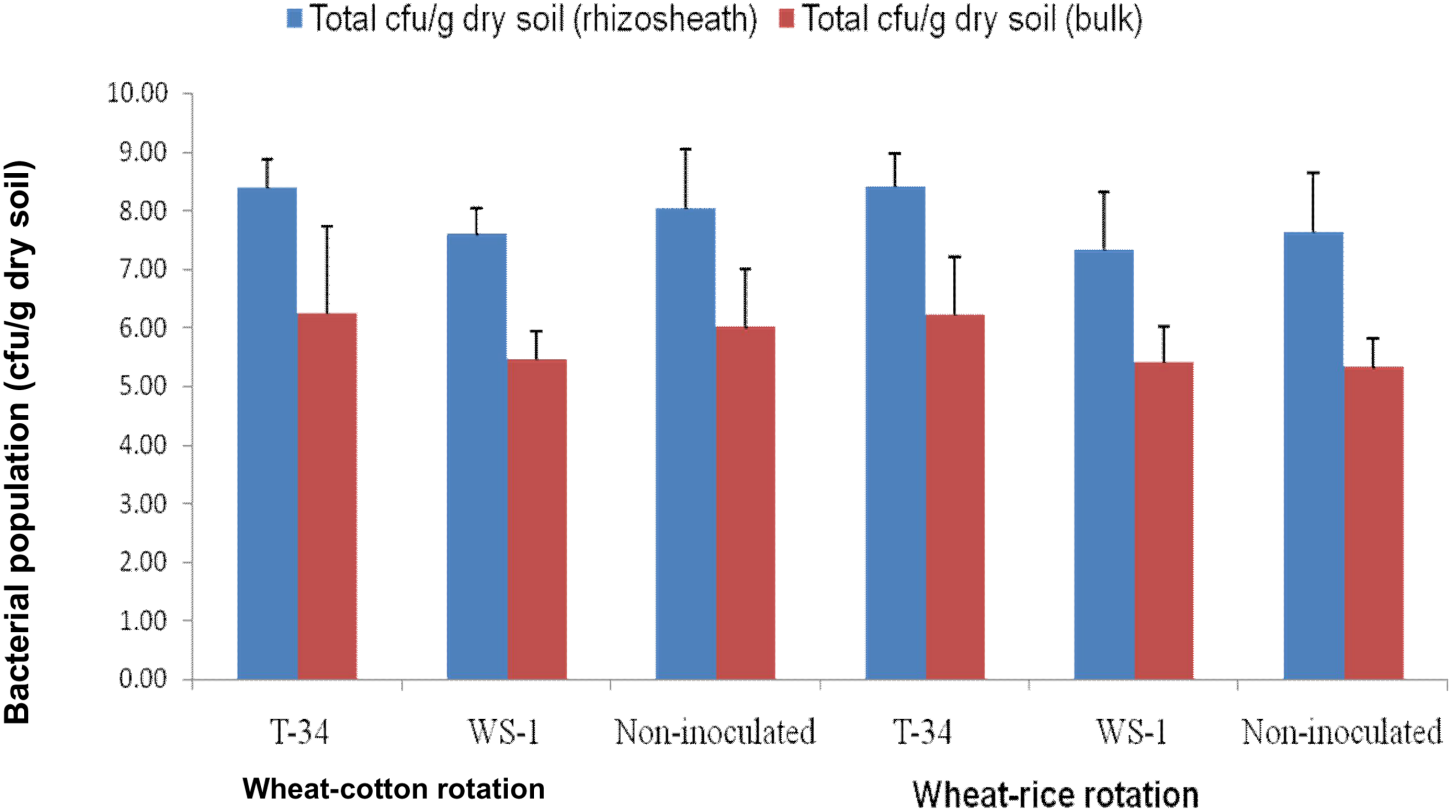
Population of culturable bacteria (cfu.g^-1^ of soil) in rhizosheath and bulk soil of wheat. Plants were grwon under wheat-rice and wheat-cotton rotation, and inoculated with *Azospirillum* sp. WS-1, *Bacillus* sp. T-34 or non-inoculated. Bacterial population sizes were measured in rhizosheath and bulk soil samples by plating of serial dilutions on LB agar. Log values were calculated for colony forming units (cfu). Given values are the mean of 3 replications.

In order to comprehensively assess the diversity and identity of bacteria in wheat rhizosheaths under both rotations, after 35 DAS, we used cultivation on LB medium, as well as pyrosequencing of PCR-amplified 16S rRNA gene fragments. Four morphologically different colony types, A, B, C and D (Fig 4A and 4B), were dominantly present along with other less abundant colony types in all plated samples of rhizosheath soil. The ranges of colony type A(log 2–4 cfu.g^-1^ of dry soil), colony type B(log 4–7 cfu.g^-1^ of dry soil), colony type C (log 4–7 cfu.g^-1^ of dry soil) and colony type D (log 2–7 cfu.g^-1^ of dry soil) were detected under both the crop rotations (Fig 4A and 4B). cfu of colony types A, C and D were highest in the rhizosheath of wheat grown under wheat-cotton rotation while the number of type B colonies was highest in the rhizosheath of wheat grown under wheat-rice crop rotation. Among the treatments maximum total cfu g^-1^ dry soil (>10^8^ cfu.g^-1^ dry soil) was obtained in the rhizosheath of plants inoculated with *Bacillus* sp. T-34 strain in both rotations (Fig 4A and 4B). The total number of cfu obtained on LB medium for bulk soil suspensions was also significantly (*P*<0.05) affected by inoculation of bacterial isolates (Fig 5). Bacterial population (cfu.g^-1^dry soil) was significantly higher (p<0.05) in soil inoculated with *Bacillus* sp. T-34 as compared to other treatments under both the systems. In this treatment total cfu.g^-1^ dry soil were higher (>10^8^ cfu.g^-1^ dry soil) in rhizosheath (Fig 5) compared with bulk soil (≤10^6^cfu.g^-1^ dry soil). One representative colony from each dominant colony type was identified by 16S rRNA gene sequence analysis. Colony type A was identified as *Bacillus* sp. (isolate PA), colony type B as *Enterobacter* sp. (isolate WP-8), colony type C as *Arthrobacter* sp. (isolate WK2T) and colony type D as *Acinetobacter* sp. isolate WS-1D (Table 4).

**Table 4:** Identification of bacterial isolates on the basis of 16S rRNA gene sequence analysis.

Isolate	Organism identified	Accession number	Closest type strain in NCBI data base	*16S rRNA* identity(%)
*WS-1D	*Acinetbacter*	HE661618	*A*.*calcoaceticus* DSM 30006T (AJ633632)	99
*WB-3P5	*Acinetobacter*	HE661628	*A*.*calcoaceticus* DSM 30006T (AJ633632)	99
**T-34B	*Agromyces*	HE661617	*A*.*indicus* type strainNIO-1018 (NR108908)	100
**T-26	*Agrobacterium*	HE661623	*A*.*tumefaceins* NCPPB2437 T (D01256)	99
*WK2T	*Arthrobacter*	HE661609	*A*. *scleromae* YH-2001T (AF330692)	97
**WS-1	*Azospirillum*	HE977616	*A*. *brasilense* SP7 ATCC 29145(NR119100)	98
**AzT-1	*Azospirillum*	HE977617	*A*. *brasilense* SP7 ATCC 29145(NR119100)	98
**AzT-2	*Azospirillum*	HE977618	*A*. *brasilense* SP7 ATCC 29145(NR119100)	98
**PA	*Bacillus*	HE661616	*B*. *subtilis* DSM 10T (AJ276351)	99
*PC	*Bacillus*	HE661620	*B*. *subtilis* DSM 10T(AJ276351)	99
**T-21	*Bacillus*	HE661621	*B*. *aquimaris* TF-12T (AF483625)	97
*T-22	*Bacillus*	HE661622	*B*. *subtilis* DSM 10T (AJ276351)	100
*T-34	*Bacillus*	HE817770	*B*. licheniformis ATCC 14580(NC006270)	98
**T-41	*Enterobacter*	HE661629	*E*. *hormaechei* EN-562T (AJ853890)	95
**T-42	*Enterobacter*	HE661630	*E*. *ludwigii* EN-119T (AJ853891)	98
*WP-8	*Enterobacter*	HE661608	*E*. *hormaechei* EN-562T (AJ853890)	94
**T-2	*Microbacterium*	HE661605	*M*. *martypicum* DSM 12512T (AM181506)	99
*WNT	*Microbacterium*	HE661631	*M*. *aurantiacum* DSM 12506T(AM182159)	99
*WP-5	*Pantoea*	HE661627	*P*. *conspicua* LMG 24534 (EU216737)	97
**T-27	*Pseudomonas*	HE661611	*P*. *poae* DSM 14936T (AJ492829)	99
*NN-25	*Pseudomonas*	HE661612	*P*. *poae* DSM 14936T (AJ492829)	98
*NN-4	*Pseudomonas*	HE661613	*P*. *mendocina* ICMP13540T (AJ308310)	98
*WP-1	*Pseudomonas*	HE661625	*P*. *poae* DSM-14936T (AJ492829); *P*. *congelans* DSM 14939T (AJ492828)	99
**T-20	*Rhodococcus*	HE661614	*R*. *erythropolis* DSM 43066T (X79289)	97
**T-5	*Sphingobacterium*	HE661610	*S*. *faecium* type strain 15299T (AB680829)	98
**NN-24	*Xanthomonas*	HE661615	*X*. *perforans*DSM 18975T (FR749910)	97

*Bacterial isolates obtained from wheat-cotton rotation

**Bacterial isolates obtained from wheat-rice rotation

16S rRNA gene was amplified from DNA of 26 bacterial isolates and PCR products were sequenced. The 16S rRNA sequences obtained were trimmed (Bio-Edit 7.1) identified using BLAST at the National Centre for Biotechnology Information web site (www.ncbi.nlm.nhi.gov) [27], and submitted to EMBL.

In total, we obtained 26 morphologicallydifferent bacterial isolates (morphotypes) from the rhizosheath of wheat grown under wheat-cotton and wheat-rice rotation, including above-mentioned representatives of the four most dominant colony types. From wheat-rice rotation, 12 bacterial isolates were obtained and 14 bacterial isolates were isolated from wheat-cotton rotation. These bacterial isolates were identified using 16S rRNA gene sequence analysis (Table 4). For most isolates we observed 97–99% similarity with the 16S rRNA gene sequences of previously cultured bacteria belonging to 13 different bacterial genera from the phyla *Firmicutes*, *Proteobacteria* and *Actinobacteria*. The genera represented by multiple strains were *Bacillus* (5 isolates), *Pseudomonas* (4 isolates), *Enterobacter* (3 isolates), *Azospirillum* (3 isolates) *Acinetobacter* (2 isolates),and *Microbacterium* (2 isolates). The remaining isolates showed close relatedness to genera *Agromyces*, *Agrobacterium*, *Arthrobacter*, *Pantoea*, *Rhodococcus*, *Sphingobacterium* and *Xanthomonas*. Among the isolates only two i.e. T-41 and WP-8 showed relatively low sequence similarity (95% and 94%, respectively) to the sequence of *Enterobacterhormaechei* EN-562T.

Phylogenetic trees were constructed using 16S rRNA gene sequences of the bacterial isolates along with related sequences in the NCBI data base (supplementary material). The phylogenetic tree constructed for *Acinetobacter* and *Pseudomonas* strains indicated clustering of the isolates WS-1D and WB-3P5 with *Acinetobacter*spp. and *Acinetobactercalcoaceticus*, respectively. The isolates NN-4 and NN-25 formed cluster with *Pseudomonasmendocina* and *P*. *poae*, respectively. The remaining two isolates WP-1 and T-27 of *Pseudomonas* joined cluster with *P*. *tolaasi* in the tree S1 Fig. All the three isolates belonging to genus *Azospirillum* formed cluster with *A*. *brasilense* strains S2 Fig. A phylogenetic tree was constructed to accommodate isolates belonging to genera *Bacillus*, *Arthrobacter* and *Rhodococcus* S3 Fig. Three isolates (T-22, PC and PA) formed cluster with *Bacillussubtilis* strains. The isolates T-34 and T-21 formed cluster with *B*. *licheniformis* and *B*. *aquaemaris* strains, respectively. In this tree one isolate formed cluster with *Arthrobacterscleromae* and one with *Rhodococuserythropolis* strains. One isolate (WNT) identified as *Microbacterium* clustered with strains of *M*. *aurantiacum* and the other T-2 with strains of *M*. *myritypicum* S4 Fig. The isolate T-26 formed cluster with *Agrobacterium tumefaceine* strains S5 Fig. The isolate T-34B clustered with *Agromyces indicus* strains S6 Fig. The bacterial isolate T-5 is present in a cluster containing *Sphingobacterium faceium* strains S7 Fig. Phylogenetic tree for *Xanthomonas* showed the presence of isolate NN-24 in cluster of *Xanthomonas* spp. S8 Fig. The phylogenetic tree constructed for *Pantoea* S9 Fig. and *Enterobacter* S10 Fig. strains indicated clustering of the isolates WP-5 with *Pantoea agglomerans* and T-41, T-42 and WP-8 with *Enterobacter* strains respectively.

To complement cultivation-based analyses, bacterial diversity was determined by 454-pyrosequencing analysis of 16S rRNA gene fragments amplified from rhizosheath DNA. For this purpose, rhizosheath soil samples were collected in duplicate (from two plots) from different treatments (*Bacillus* T-34, *Azospirillum* WS-1, non-inoculated control). From these 12 samples a total of 57,638 high quality sequences were obtained with an average read length of 319 bp after trimming of primer sequences. Of these sequences, 46,971 reads were classifiable when OTUs were defined at 97% similarity level, with a range of 3149–7513 reads/sample.

Community richness (*d*), Shannon diversity index (*H*) and evenness (*E*_*H*_) were calculated. Community richness was higher (*d* = 2714.9 and 1715.9) in DNA obtained from rhizosheath of non-inoculated plants under wheat-cotton rotation as compared to those of inoculated treatments. Under wheat-rice rotation, community richness was higher in rhizosheath soil DNA of *Azospirillum*-inoculated plants (Table 5). Shannon diversity index (*H* = 0.37 and 0.35) was higher in *Bacillus* sp. T-34 inoculated rhizosheath soil sample compared to that of non-inoculated (*H* = 0.23 and 0.34) rhizosheath soil sample under wheat-cotton rotation. The same trend was observed under wheat-rice rotation. Evenness (*E* = 0.052 and 0.049) was also higher in communities obtained from rhizosheath soil DNA of plants inoculated with *Bacillus* under wheat-cotton as well as wheat-rice rotation (*E* = 0.023 and 0.038) when compared with non-inoculated plant rhizosheath. Similarly, higher fraction (%) of sequences related to PGPR genera were detected in rhizosheath soil DNA of inoculated plants under both the crop rotations (Table 5).

**Table 5:** Species richness, diversity and evenness in bacterial communities of wheat rhizosheath under wheat-rice and wheat-cotton rotation.

Treatments		Total number of sequences	Community richness (*d*)	Evenness (E_H_)	Shannon diversity index (H)	Fraction of sequences (%) most closely related to PGPR genera
**Wheat-cotton rotation**	***Azospirillum* sp. WS-1 R1**	2831	1256.2	0.036	0.25	0.80
	***Azospirillum* sp. WS-1R2**	3452	1432.9	0.051	0.36	0.82
	***Bacillus* sp. T-34 R1**	2546	1143.7	0.052	0.37	0.78
	***Bacillus* sp. T-34R2**	3843	1606.9	0.049	0.35	0.79
	**Non-inoculated R1**	5985	2714.9	0.032	0.23	0.52
	**Non-inoculated R1**	4079	1715.9	0.048	0.34	0.51
**Whet-rice rotation**	***Azospirillum* sp. WS-1 R1**	4321	2146.2	0.021	0.15	0.71
	***Azospirillum* sp. WS-1 R2**	4152	1840.6	0.036	0.25	0.72
	***Bacillus* sp. T-34 R1**	4176	2029.7	0.023	0.16	0.72
	***Bacillus* sp. T-34 R1**	3652	1607.1	0.038	0.26	0.73
	**Non-inoculated R1**	4270	2064.1	0.018	0.13	0.56
	**Non-inoculated R2**	3339	1423.8	0.014	0.10	0.53

Nucleotide sequences (319 bp) obtained from pyrosequencing using barcodes was statistically analyzed.

**Fig 6:**
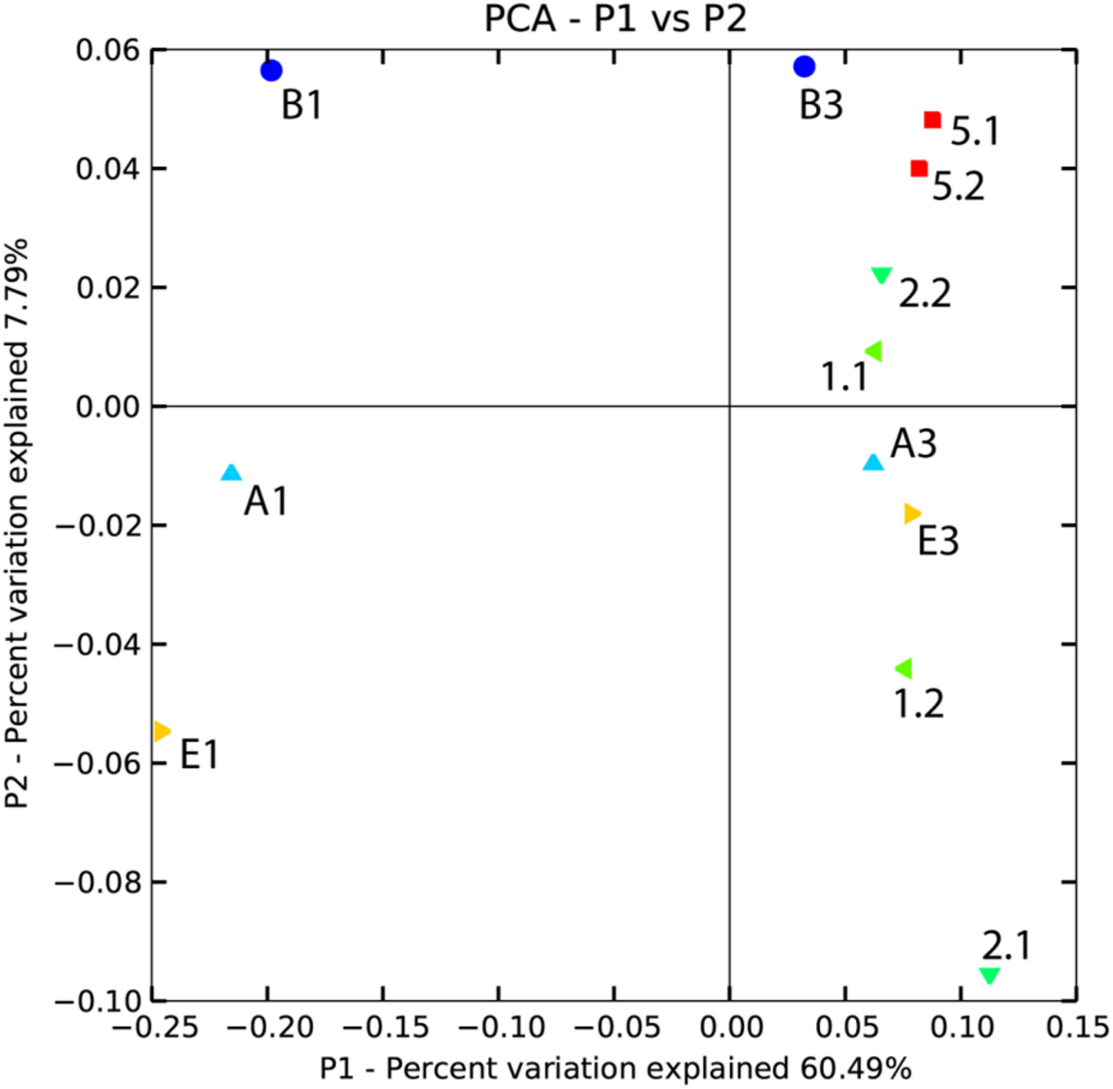
PCoA plot of weighted UniFrac distances of rhizosheath soils samples from wheat under wheat-cotton and wheat-rice rotation. Wheat-cotton rotation: rhizosheath soil samples inoculated with *Bacillus* sp. T-34 (A1 and A3); Rhizosheath soil samples inoculated with *Azospirillum* sp. WS-1 (B1 and B3) and rhizosheath soil samples of non-inoculated plants (E1 and E3). Wheat-rice rotation:: rhizosheath soil samples inoculated with *Bacillus* sp. T-34 (1.1 and 1.2); Rhizosheath soil samples inoculated with *Azospirillum* sp. WS-1 (2.1 and 2.2) and rhizosheath soil samples of non-inoculated plants (5.1 and 5.2).

Principal Coordinate Analysis (PCoA) of pyrosequencing data was performed to assess potential differences in the composition of the different bacterial communities (Fig 6). Overall, the first two principal coordinates explained 60.49% and 7.79% of the variation in the compositional data, and revealed clear differences between bacterial communities in both crop rotations as well as inoculated and non-inoculated rhizosheath. Rhizosheath soil B1 (*Azospirillum* sp. WS-1 inoculation under wheat-cotton rotation), rhizosheath soil A1 (inoculated with *Bacillus* sp. T-34 under wheat-cotton rotation) and rhizosheath soil E1 (non-inoculated under wheat-cotton rotation) were found separated from the rest of the samples, including corresponding duplicates, along the first principal coordinate axis (Fig 6). For other samples, duplicates were found separated mostly along the second principal coordinate, with samples 5.1 and 5.2 (non-inoculated, wheat-rice rotation) being most closely related.

**Fig 7:**
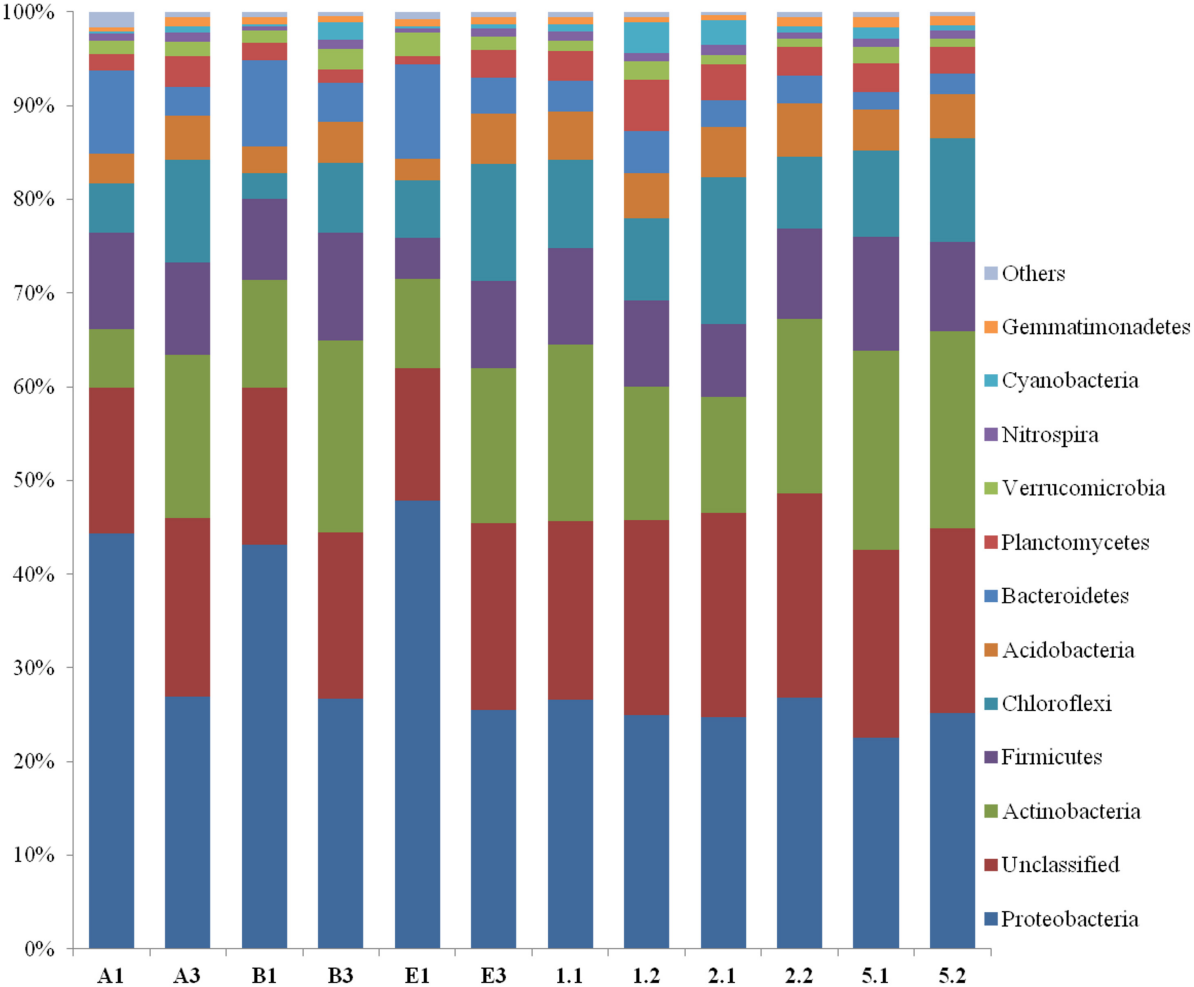
Relative abundance of bacterial phyla detected in rizosheath based on 454 pyrosequencing of 16S rRNA gene. All phyla contributing to less than 1% of the total bacteria were grouped as ‘Other’. Wheat-cotton rotation: rhizosheath soil samples inoculated with *Bacillus* sp. T-34 (A1 and A3); Rhizosheath soil samples inoculated with *Azospirillum* sp. WS-1 (B1 and B3) and rhizosheath soil samples of non-inoculated plants (E1 and E3). Wheat-rice rotation:: rhizosheath soil samples inoculated with *Bacillus* sp. T-34 (1.1 and 1.2); Rhizosheath soil samples inoculated with *Azospirillum* sp. WS-1 (2.1 and 2.2) and rhizosheath soil samples of non-inoculated plants (5.1 and 5.2).

Rhizosheath bacterial communities in both crop rotations exhibited similar overall patterns of relative abundance of the major groups at the phylum level (Fig 7). In both rotations, *Proteobacteria* were the most predominant phylum, being 25.1% and 35.7% of the total detected sequeces in wheat-cotton and wheat-rice rotation, respectively (Fig 7). Comparison of the relative abundance of different bacterial phyla in both rotation systems showed that *Proteobacteria*, *Bacteriodetes* and *Verrucomicrobia* were present at higher relative abundance in wheat-rice rotation, where as *Actinobacteria*, *Firmicutes*, *Chloroflexi*, *Acidobacteria*, *Planctomycetes* and *Cyanobacteria* were more abundant in wheat-cotton rotation (Fig 7).

**Fig 8:**
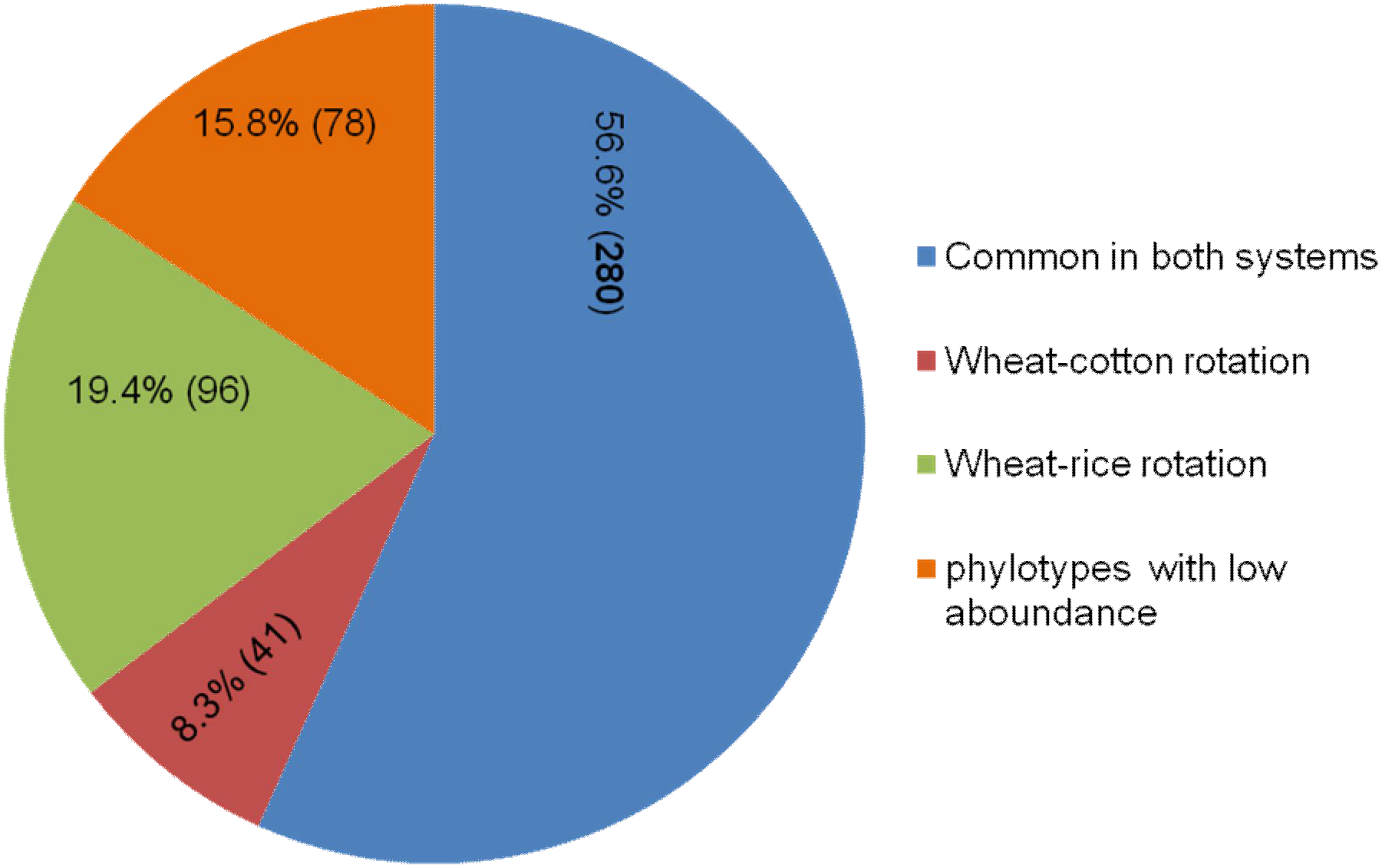
Distribution of phylotypes identified through pyrosequencing of 16S rRNA gene fragments. Distribution of phylotypes defined at 97% sequence similarity threshold Numbers in parentheses indicate numbers of phylotypes found. Rare phylotypes with low relative abundance were not consistently detected in any of the sample types.

It was also observed that out of 495 different phylotypes detected, 280 (56.6%) were common in both crop rotations, whereas 96 (19.4%) were only found in wheat-rice rotation and 41 (8.3%) were only present in wheat-cotton. A total of 78 (15.7%) OTUs were only rarely (i.e. represented by less than 5 sequences/sample) found in the samples (Fig 8).

Out of the total 46,971 sequences, 7,835 sequences (16.7%) showed 97% or higher similarity with 16S rRNA gene sequences of in total 32 genera previously described to have PGPR activity (Table 6). Comparing the treatments under wheat-cotton rotation, of the total sequences obtained from *Bacillus* sp. T-34 inoculated rhizosheath soil sample, 25 genera (0.78% of the total sequences) with known PGPR activity were found. The highest number of PGPR genera (0.80% of total sequences) was detected in rhizosheath soil inoculated with *Azospirillum* sp. WS-1 (data not shown). Among PGPR, the most dominant genera detected commonly in both cropping systems were *Bacillus*, *Arthrobacter*, *Azospirillum*, *Paenibacillus*, *Pseudomonas*, *Gordonia*, *Cyanobacterium* and *Rhizobium*. However, the sequences belonging to diazotrophic genus *Delftia* were detected only in wheat-rice cropping system (Table 6). The sequences related to some important diazotrophic genera like *Rhizobium*, *Bradyrhizobium*, *Cyanobacteria* and *Flavobacterium* were more aboundant in rhizosheath of wheat under wheat-rice rotation as compared to wheat-cotton rotation. The sequences belonging to the important PGPR genera like *Arthrobacter*, *Bacillus* and *Gordonia* were relatively higher under wheat-cotton crop rotation (Table 6). *Bacillus*, *Arthrobacter*, *Azospirillum* and *Pseudomonas* were also frequently retrieved from wheat rhizosheaths by cultivation as described above. Some nitrogen fixing genera like *Azospira*, *Azonexus* and *Azoarcus* were specifically present in wheat rhizosheaths under wheat-rice rotation but we were unfortunately unable to isolate these beneficial bacteria.

**Table 6:** Number of sequences related to genera identified by16S rRNA gene pyrosequencing previously found to include PGPRs.

Genus	Wheat-cotton rotation	Wheat-rice rotation	Genus	Wheat-cotton rotation	Wheat-rice rotation
***Achromobacter*^abc^**	19	90	***Gordonia*^b^**	160	83
***Acinetobacter*^abc^**	1	129	***Herbaspirillum*^ac^**	69	81
***Arthrobacter*^bc^**	808	439	***Microbacterium*^bc^**	6	20
***Azoarcus*^a^**	3	3	***Ochrobactrum*^bc^**	2	21
***Azohydomonas***	34	10	***Optitutae*^a^**	2	8
***Azospirillum*^abc^**	24	21	***Paenibacillus*^ab^**	129	105
***Azotobacter*^ac^**	18	7	***Pantoea*^abc^**	3	1
***Bacillus*^abc^**	1185	786	***Phyllobacterium*^b^**	56	69
***Bradyrhizobium*^a^**	37	62	***Pseudomonas*^abc^**	85	455
***Burkholderia*^abc^**	142	310	***Porphyrobacter*^bc^**	23	57
***Cynobacteria*^a^**	10	57	***Proteus*^ab^**	2	0
***Delftia*^ab^**	0	15	***Rhizobium*^abc^**	41	373
***Enterobacter*^abc^**	1	1	***Rhodobacter*^b^**	0	6
***Erythrobacter*^c^**	5	26	***Serratia*^b^**	4	2
***Escherichia*^b^**	5	4	***Skermanella***	177	170
***Flavobacterium*^b^**	23	188	***Klebsiella*^abc^**	2	3

Nucleotide sequences (319 bp) obtained from pyrosequencing using barcodes was statistically analyzed. Sequences that showed >97% similarity with previously reported PGPR genera were grouped. Letter ‘a’ represents nitrogen fixing genera, ‘b’ represents IAA producing genera and ‘c’ represents phosphate solubilizing bacterial genera [18], [19], [20], [21], [42], [43], [44], [46], [47], [51], [52], [53], [54], [55], [56], [57], [58], [59],

## Discussion

Rhizosheath formation was observed around the roots of wheat grown under wheat-rice and wheat-cotton based rotations in our study. Rhizosheaths formed around the roots of wheat are specialized structures to increase the water holding capacity of soil, nutrient uptake and also harbour bacteria with plant beneficial traits [8], [9]. Moisture content of rhizosheaths under wheat-cotton and wheat-rice rotation was respectively 4 and 2% units higher than that of the surrounding bulk soil. Our data indicated that moisture contents in rhizosheath of inoculated plants were higher as compared to non-inoculated control plants (Table 2). This indicates the role of roots and activity of inoculated bacterial strains of *Bacillus* and *Azospirillum* in water holding capacity of rhizosheaths originating from the improved plant growth and enhanced root exudation. Strong positive relationship was observed between root exudates (organic acids) and moisture contents of the rhizosheath (R^2^ = 0.5 and 0.99 under wheat-cotton and wheat-rice rotation, respectively with regression equation y = 0.016x+0.3 and y = 0.005x-0.001). Previous studies indicated that strains of *Bacillus* and *Azospirillum* produced organic acids [20] which may keep the soil moist for longer time. Our results are similar to those of previous studies that showed that the length and density of the rhizosheath depends on the moisture content of soil [8], [9], [10]. Optimum moisture content around the roots increases enzymatic activity, especially acid phosphatase [40], which improves the phosphorous availability to plants, consequently increasing crop growth [41], [42]. Soil moisture content also influences bacterial community structure [40], which defines beneficial interaction of bacteria with the roots and improves the soil fertility level and yield of plants. Dry weight of soil attached to the roots as rhizosheaths was higher under wheat-cotton rotation compared to wheat-rice rotation (Table 3), which could be related to differences in the physico-chemical characteristics of the soil in the two systems. To that end, the soil in the wheat-rice rotation had a higher salt concentration and a lower amount of organic matter.

In our study root length, and root and shoot dry weight increased with the application of bacterial strains of the genera *Bacillus* and *Azospirillum* over non-inoculated plants (Table 3). Our data indicated that root exudates (organic acids concentration) dry weight of soil attached with roots, root length, root and shoot dry weight were significantly higher in inoculated plants when compared with that obtained from non-inoculated control plants (Table 3). Correlation coefficient indicated that positive correlation exist between the root exudates and root length (R^2^ = 0.96 and 0.80 respectively under wheat-cotton and wheat-rice rotation), root exudates and shoot dry weight (R^2^ = 0.99 under both the crop rotations), root exudates and root dry weight (R^2^ = 0.99 and 0.58 under wheat-cotton and wheat-rice rotation, respectively). In our previous studies [20] both the bacterial strains *Azospirillum* sp. WS-1 and *Bacillus* sp. T-34 tested in the present study as inoculants have been shown to produce growth hormone Indol-3-acetic acid (IAA), solubilize phosphorous and improved the growth of wheat. Members of both these genera have previously been reported to exhibit plant growth promoting traits [9], [18], [19], [20], [21]. All agronomic practices affecting plant growth like irrigation, seed rate, fertilizer application and weed control were kept the same in all inoculated and non-inoculated control treatments. Therefore improved growth of inoculated plants was attributed to growth promoting traits of the inoculated bacterial strains. However, studies focusing on localization of inoculated bacteria in the rhizosphere (e.g. FISH), use of genetically marked strains or cultivation on strain-specific growth media may further strengthen the observation that the inoculated bacteria successfully colonized the rhizosphere (rhizosheath) to cotribute to plant growth. Higher nitrogen contents in dry plant material of *Azopsirillum* sp. WS-1 inoculated plants and high phosphorous contents in the dry plant material of *Bacillus* sp. T-34 inoculated plants as compared to non-inoculated plants under both the crop rotations suggeted that bacterial inoculation may have contributed to increased nitrogen and phosphorous uptake and accumulation in plant. In the present experiment better plant growth (root length, root and shoot dry weight) was observed under wheat-cotton rotation compared to wheat-rice rotation (Table 3). According to pyrosequencing data, the higher number of sequences related to PGPR genera (*Azospirillum*, *Arthrobacter*, *Bacillus*, *Enterobacter* and *Pseudomonas*) was detected in rhizosheaths of wheat plants grown in wheat-cotton rotation. More favourable soil characteristics with respect to soil organic matter, available nutrient status, moisture contents and low salt concentration were found in soil under wheat-cotton rotation compared to wheat-rice rotation. All these factors may have contributed to the formation of better rhizosheath under wheat-cotton rotation, which helps in better nutrient acquisition and water holding capacity, promoting plant growth [8], [16].

Root exudates are released into the rhizosphere as a result of rhizodeposition and play a major role in the maintenance of root-soil and root-microbe contacts [15], [16]. In our study organic acids including acetic acid, citric acid, oxalic acid and malic acid, and the two sugars sucrose and glucose, were detected in root exudates collected from wheat rhizosheath under wheat-rice and wheat-cotton based cropping systems. The presence of organic acids and sugars in the rhizosphere of different crops has been reported previously [16], [43]. In our study we observed higher amounts of organic acids in the rhizosheaths formed under wheat-cotton rotation compared to wheat-rice rotation (Fig 2A and 2B). Favourable physical and chemical conditions in soil resulted in improved health of plants which enhanced root exudation and accumulation of organic acids in the well-developed rhizosheathunder wheat-cotton rotation. Root exudate accumulation, secretion and formation of rhizosheaths have been reported in various grasses [8], [9], [16]. In the present study, secretion of organic acids and sugars was also affected by bacterial inoculation. Organic acids concentration was higher in rhizosheath of plants inoculated with *Azospirillum* sp. WS-1 and *Bacillus* sp. T-34 as compared to non-inoculated plants (Fig 2A and 2B), while sugar (glucose and sucrose) concentration was lower in rhizosheaths of inoculated plants compared to non-inoculated plants (Fig 3A and 3B). This is in line with previous reports indicating that bacilli and azospirilla produce organic acids by utilizing sugars like glucose, galactose, sucrose and fructose under laboratory conditions [20], [44], [45], [46], [47]. Hence it was hypothesized that by utilizing the sugars (glucose, sucrose, galactose, fructose) present in rhizosphere [16], [48] bacteria may produce the same organic acids as in *in vitro* culture media. In the present study, organic acid secretion was also influenced by root length and root dry weight, as a higher concentration of root exudates was correlated with higher root length (R^2^ = 0.9 and 0.8 under wheat-cotton and wheat-rice rotation, respectively) and root dry weight (R^2^ = 0.99 and 0.58 under wheat-cotton and wheat-rice rotation, respectively). These results are in line with those previously reported [8], [14], [49].

Our results showed that the numbers of culturable bacteria were higher in rhizosheaths inoculated with *Bacillus* sp. T-34 strain under wheat-cotton rotation compared to wheat-rice rotation when compared with *Azospirillum* sp. WS-1 inoculated plants and non-inoculated control plants (Fig 4A and 4B). Furthermore, dry weight of soil attached with roots as rhizosheath and moisture contents were higher in *Bacillus* sp. T-34 inoculated plants compared to *Azospirillum* sp. WS-1 inoculated plants and non-inoculated control plants. Members of the genus *Bacillus* as PGPR (P-solubilizers and growth hormone producers) have been frequently reported [18], [19], [20], [21]. Strain *Bacillus*T-34 is well known reported PGPR [20] for wheat. It is tempting to speculate that its inoculation to wheat improved the plant health and growth, which consequently resulted in secretion of higher amount of root exudates (organic acids) compared to *Azospirillum* sp. WS-1 inoculated plants and non-inoculated control plants, resulting in creation of more favourable conditions for different bacteria to come in contact with roots of *Bacillus* sp. T-34 inoculated plants. It has been previously reported that the bacterial density and diversity was influenced by crop rotation, root exudates, moisture contents, soil nutrient status and rhizosheath formation [1], [6], [16]. Furthermore, we observed that the number of culturable bacteria (cfu.g^-1^ dry soil) was higher in rhizosheath compared with bulk soil (Fig 5). The reasons for higher cfu.g^-1^ dry soil in rhizosheath are the presence of root exudates (organic acids and sugars) and the higher moisture contents, which attract bacteria to come in contact with rhizosheath compared with bulk soil. This indicated that high moisture contents and root exudates (organic acids and sugars) created more favourable conditions for bacteria like *Arthrobacter*, *Acinetobacter*, *Bacillus* and *Enterobacter* to associate with rhizosheath rather than in bulk soil and, therefore, the population of these bacteria was increased in rhizosheath over bulk soil.

We isolated and identified strains of *Arthrobacter*, *Acinetobacter*, *Azospirillum*, *Bacillus*, *Enterobacter*, *Microbacterium*, *Pantoea*, *Pseudomonas*, *Rhodococcus*, *Sphingobacterium* and *Xanthomonas*. Isolation of bacteria, especially *Bacillus* spp., from the rhizosheaths of different crops has been reported [11]. Isolation of *Azospirillum*, *Bacillus* and *Enterobacter* strains from the rhizosphere of wheat, rice and sugarcane has been reported previously from the same cropping area of Pakistan [20], [22], [50]. The isolates obtained in the present study included important PGPR like *Azospirillum* and *Bacillus*, reinforcing previous studies that root exudates (organic acids and sugars) attract beneficial bacteria to come in contact with the rhizosphere, leading to increased abundance and diversity of bacteria [15], [16]. The rhizosheath environment extends the root band that supports microbial activity and consequently increases the plant-microbe interaction [11].

In the present study, sequence analysis of the 16S rRNA gene pool directly amplified from rhizosheath DNA indicated the presence of 32 genera for which isolates with PGPR activities (nitrogen fixation, P solubilization and IAA production) have been reported (Table 6). The most dominant PGPR genera were *Bacillus*, *Arthrobacter*, *Azospirillum*, *Paenibacillus*, *Pseudomonas*, *Gordonia*, *Cyanobacterium* and *Rhizobium*. Sequences related to nitrogen fixing genera like *Azospirillum*, *Azoarcus*, *Azotobacter*, *Bacillus*, *Cyanobacterium*, *Enterobacter* and *Rhizobium* were detected in rhizosheath soil. Nitrogen fixation by these genera has been widely described [46], [51], [52], [53], [54], [55], [56], [57],[58]. Similarly, sequences related to genera presenting phosphate solubilizing activity like *Arthrobacter*, *Azospirillum*, *Bacillus*, *Enterobacter* and *Paenibacillus* were detected. Phosphate solubilization by these bacterial genera has been well documented [18], [19], [20], [21], [47], [59]. Predominant phylotypes obtained by cultivation were also detected in the rhizosheath by pyrosequencing.

Ten bacterial phyla were detected in our data under two cropping systems. The most abundant phyla were the *Proteobacteria* and *Actinobacteria* and less dominant were the *Acidobacteria*, *Planctomycetes*, *Verrucomicrobia* and *Cyanobacteria* (Fig 7). The detection of these phyla has been reported through metagenomics studies on different soils [60], [61]. Interestingly, *Firmicutes* (bacilli) were less dominant in these published data but were abundantly present in both crop rotations in our study. This may be due to suitable soil conditions for growth and partially due to fact that *Bacillus* spp. based biofertilizers have been used frequently in our soils, leading to their increased abundance. This may also be due to the fact that other studies were conducted on bulk soil whereas we reported the bacterial communities in rhizosheath.

In our data about 57% of all detected genera were commonly found in both rotations (Fig 8). Results obtained by Roesch *et al*. (2007) and Fulthorpe *et al*. (2008) [61], [62] showed that some genera (30%) were commonly found in distantly sampled soils. This may also be due to the reason that the genera like *Bacillus*, *Azospirillum*, *Pseudomonas* and *Enterobacter* which have shared OTU can survive with the condition prevailing in rhizosheath under both the crop rotations. However, *Rhizobium* a well known nitrogen fixing was only found in rhizosheath under wheat-rice rotation because the soil conditions under this system favours the survial of these bacteria. Our data showed that out of 495 phylotypes, 19.4% were present only in wheat-rice rotation and 8.3% were uniquely present in wheat-cotton rotation, which might be attributable to the observed differences in physico-chemical characteristics of the corresponding soils. It has been reported that bacterial composition and abundance are influenced by soil moisture contents, crop rotation, tillage practices, nature and amount of root exudates and crop species [1], [6], [7], [16].

It was observed that percentage of sequences related to genera with known PGPR activity was higher in inoculated rhizosheath samples (0.80% of the total sequences from *Azospirillum* sp. WS-1 inoculated rhizosheath and 0.78% of the total sequences from *Bacillus* sp. T-34 inoculated rhizosheath) compared to non-inoculated rhizosheath soil (0.52%). This suggested that the inoculation of PGPR stimulated growth of indigenous beneficial bacteria to come in contact with the rhizosheath of plants and contribute to the overall better performance of crop. Many of the bacterial genera identified by pyrosequencing in the present study are known to harbor PGPR strains. Diversity and evenness among the inoculated rhizosheath bacterial communities were higher as compared to that of non-inoculated rhizosheath under wheat-cotton rotation and wheat-rice rotation. Well-developed rhizosheath,optimum moiture contents, higher amount of organic acids andhigher root length of inoculated plants under wheat-cotton rotationattracted the diverse bacteria towards roots and provided favourable conditions for multiplication [1], [6], [7], [16]. Principal Coordinate Analysis (Fig 6) showed the rhizosheath soil samples from inoculated and non-inoculated plants under both the crop rotations present in different coordinates and formed distinct clusters. As discussed in Material and Methods, rhizosheath soil samples were collected (five times) from each inoculated and non-inoculated plots and these five samples were mixed to get one pooled sample. Actually one sample from each replicate is a pool of five samples. Similarly DNA was extracted from pooled samples and processed therefore number of replicates were sufficient. To minimize the replication error in bacterial community structure of rhizosheath, numbers of samples were collected from each plot. However, the separation of some samples duplicates indicates that in future studies many more samples should be analyzed to get more precise information on bacterial community structure. It means that crop rotation and inoculation of bacteria play a role in shaping microbial community structure and diversity. It can be assumed that bacterial communities associated with rhizosheath of wheat under wheat-rice rotation were different from those associated with rhizosheath under wheat-cotton rotation at least partly because of the prolonged reducing conditions in rice cropping. Our results confirmed findings reported by previous studies [1], [6], [7], [16] which reported that crop rotation, moisture contents, tillage practices and nature of root exudates affect the microbial community structure.

Some nitrogen fixing genera like *Azospira*, *Azonexus* and *Azoarcus* were specifically present in wheat rhizosphere under wheat-rice rotation. Particularly the nitrogen-fixing genus *Azoarcus* was present in wheat rhizosheath under wheat-rice rotation. The first representative of this genus was isolated from rhizosphere of kalar grass in Punjab province of Pakistan [56]. Diverse nitrogen fixing genera are frequently present in Pakistan soils as confirmed in our studies. Unfortunately, current efforts towards isolation of representative species/strains of some of these genera, especially *Azoarcus*, have been unsuccessful. However, using more appropriate culturing conditions (growth medium, incubation temperature and laboratory conditions), the bacteria belonging to these important diazotrophic genera may be isolated and their nitrogen fixing potential may be explored for production of biofertilizers.

We obtained only 26 morphologically different bacterial isolates belong to 13 different bacterial genera in culture-dependent studies. Among these, the strains belong to the genera *Acinetobacter*, *Arthrobacter* and *Pantoea* were only obtained from wheat-cotton rotation. Strains belong to the genera *Agromyces*, *Agrobacterium*, *Azospirillum*, *Rhodococcus*, *Sphingobacter* and *Xanthomonas* were specifically isolated from wheat-rice crop rotation. Data indicated that the strains belonging to genera *Bacillus*, *Enterobacter*, *Microbacterium* and *Pseudomonas* were isolated from both the crop rotations. In the culture-independent studies, 57,638 high quality sequences were obtained with an average read length of 319 bp after trimming of primer sequences. Of these sequences, 46,971 reads were classifiable when OTUs were defined at 97% similarity level, with a range of 3149–7513 reads/sample. More diverse bacterial community with 495 different phylotypes were obtained in culture-independent technique. All the bacterial genera identified in culture dependent studies were also detected in culture-independent studies from both the crop rotations. This indicated that the genus which was specific to a particular crop rotation in culture-dependent studies was commonly detected in both the crop rotations in culture-independent technique. Previous studies reported that biosphere is dominated by microorganisms and only 0.1–10% microorganisms are culturable while the majority of microorganisms remain uncultured [2], [23], [24]. Pyrosequencing provides a huge amount of parallel sequences obtained from a single DNA as compared to traditional culture-dependent methodology [60], [61]. The results of the present study are in line with the previous studies that culture-independent pyrosequencing provides a clear picture of the soil bacterial community [2], [60], [61].

In the past studies focusing on isolation and characterization of plant growth promoting rhizobacteria from the rhizosphere of various crops, have been done in Pakistan [20], [22], [50]. However, for the first time we attempted bacterial isolation and characterization from the rhizosheath of wheat. Secondly to our knowledge this is the first study on rhizosheath microbiology conducted to investigate diversity of bacteria in two different cropping systems, simultaneously using culture-dependent as well as culture-independent DNA-based techniques.

## Conclusions

In the present study higher amounts of organic acids, moisture contents, greater root length, root and shoot dry weight was observed in well-developed rhizosheaths formed around the roots of wheat grown under wheat-cotton rotation compared with wheat-rice rotation, which in return influenced bacterial abundance and community structure. Favourable growth conditions in the rhizosheath under wheat-cotton and wheat-rice rotation increased the population of cultivable bacteria in the rhizosheath over bulk soil. Rhizosheaths harbour a broad diversity of microorganisms for which plant growth promoting activities could be predicted based on 16S rRNA gene pyrosequence analysis of rhizosheath soil DNA. At least 32 bacterial genera for which one or more plant growth promoting traits have been reported, were detected in the present study through DNA-based techniques. Furthermore, about 57% of all detected genera through pyrosequencing were commonly found in both the crop rotations. Out of 495 phylotypes deted, 19.4% were present only in wheat-rice rotation and 8.3% were uniquely present in wheat-cotton rotation.

## Supporting Information

S1 Fig Phylogenetic relationship of different strains of genus *Acinetobacter* and *Pseudomonas* on the basis of 16S rRNA gene sequences. Amplified 16S rRNA gene fragments from the isolated strains of *Acinetobacter* and *Pseudomonas* were sequenced and BLAST searched through NCBI database. Closely related sequences were downloaded and aligned using CLUSTAL X. These sequences were analyzed using neighbor-joining method. The bootstrap replicates (BS) values of 50% or greater represent well supported nodes and thus only those were retained. Type strain of *Azotobacter vinelandii* was taken as outgroup.
(TIF)

S2 Fig Phylogenetic relationship of different strains of genus *Azospirillum* on the basis of 16S rRNA gene sequences. Amplified 16S rRNA gene fragments from the isolated strains of *Azospirillum* (WS-1, AzT-1 and AzT-2) were sequenced and BLAST searched through NCBI database. Closely related sequences were downloaded and aligned using CLUSTAL X. These sequences were analyzed using neighbor-joining method. The bootstrap replicates (BS) values of 50% or greater represent well supported nodes and thus only those were retained. Type strain of *Rhodospirillum oryzae* was taken as outgroup.
(TIF)

S3 Fig Phylogenetic relationship of different strains of genus *Arthrobacter*, *Bacillus* and *Rhodococcus* on the basis of 16S rRNA gene sequences. Amplified 16S rRNA gene fragments from the isolated strains PA, PC, T-21, T-22, T-34, WK2T and T-20 were sequenced and BLAST searched through NCBI database. Closely related sequences were downloaded and aligned using CLUSTAL X. These sequences were analyzed using neighbor-joining method. The bootstrap replicates (BS) values of 50% or greater represent well supported nodes and thus only those were retained. Type strain of *Micrococcus endophyticus* was taken as outgroup.
(TIF)

S4 Fig Phylogenetic relationship of different strains of genus *Microbacterium* on the basis of 16S rRNA gene sequences. Amplified 16S rRNA gene fragments from the isolated strains WNT and T-2 were sequenced and BLAST searched through NCBI database. Closely related sequences were downloaded and aligned using CLUSTAL X. These sequences were analyzed using neighbor-joining method. The bootstrap replicates (BS) values of 50% or greater represent well supported nodes and thus only those were retained. Type strain of *Agrococcus terreus* was taken as outgroup.
(TIF)

S5 Fig Phylogenetic relationship of different strains of genus *Agrobacterium* on the basis of 16S rRNA gene sequences. Amplified 16S rRNA gene fragments from the isolated strains T-26 was sequenced and BLAST searched through NCBI database. Closely related sequences were downloaded and aligned using CLUSTAL X. These sequences were analyzed using neighbor-joining method. The bootstrap replicates (BS) values of 50% or greater represent well supported nodes and thus only those were retained. Type strain of *Mesorhizobium temperatum* was taken as outgroup.
(TIF)

S6 Fig Phylogenetic relationship of different strains of genus *Agromyces* on the basis of 16S rRNA gene sequences. Amplified 16S rRNA gene fragments from the isolated strains T-34B was sequenced and BLAST searched through NCBI database. Closely related sequences were downloaded and aligned using CLUSTAL X. These sequences were analyzed using neighbor-joining method. The bootstrap replicates (BS) values of 50% or greater represent well supported nodes and thus only those were retained. Type strain of *Agrococcus terreus* was taken as outgroup.
(TIF)

S7 Fig Phylogenetic relationship of different strains of genus *Sphingobacterium* on the basis of 16S rRNA gene sequences. Amplified 16S rRNA gene fragments from the isolated strains T-5 was sequenced and BLAST searched through NCBI database. Closely related sequences were downloaded and aligned using CLUSTAL X. These sequences were analyzed using neighbor-joining method. The bootstrap replicates (BS) values of 50% or greater represent well supported nodes and thus only those were retained. Type strain of *Pedobacter agri* was taken as outgroup.
(TIF)

S8 Fig Phylogenetic relationship of different strains of genus *Xanthomonas* on the basis of 16S rRNA gene sequences. Amplified 16S rRNA gene fragments from the isolated strains NN-24 was sequenced and BLAST searched through NCBI database. Closely related sequences were downloaded and aligned using CLUSTAL X. These sequences were analyzed using neighbor-joining method. The bootstrap replicates (BS) values of 50% or greater represent well supported nodes and thus only those were retained. Type strain of *Frateuria aurantia* was taken as outgroup.
(TIF)

S9 Fig Phylogenetic relationship of different strains of genus *Pantoea* on the basis of 16S rRNA gene sequences. Amplified 16S rRNA gene fragments from the isolated strains ofWP-5 was sequenced and BLAST searched through NCBI database. Closely related sequences were downloaded and aligned using CLUSTAL X. These sequences were analyzed using neighbor-joining method. The bootstrap replicates (BS) values of 50% or greater represent well supported nodes and thus only those were retained. Type strain of *E*.*coli* was taken as outgroup.
(TIF)

S10 Fig Phylogenetic relationship of different strains of genus *Enterobacter* on the basis of 16S rRNA gene sequences. Amplified 16S rRNA gene fragments from the isolated strains ofT-41, T-42 and WP-8 was sequenced and BLAST searched through NCBI database. Closely related sequences were downloaded and aligned using CLUSTAL X. These sequences were analyzed using neighbor-joining method. The bootstrap replicates (BS) values of 50% or greater represent well supported nodes and thus only those were retained. Type strain of *E*.*coli* was taken as outgroup.
(TIF)
