# Supplementary figures and images for: Cultivation-Based and Molecular Assessment of Bacterial Diversity in the Rhizosheath of Wheat under Different Crop Rotations
Source: PLoS One. 2015 Jun 29;10(6):e0130030. doi: 10.1371/journal.pone.0130030 (PMC4487687; doi:10.1371/journal.pone.0130030)

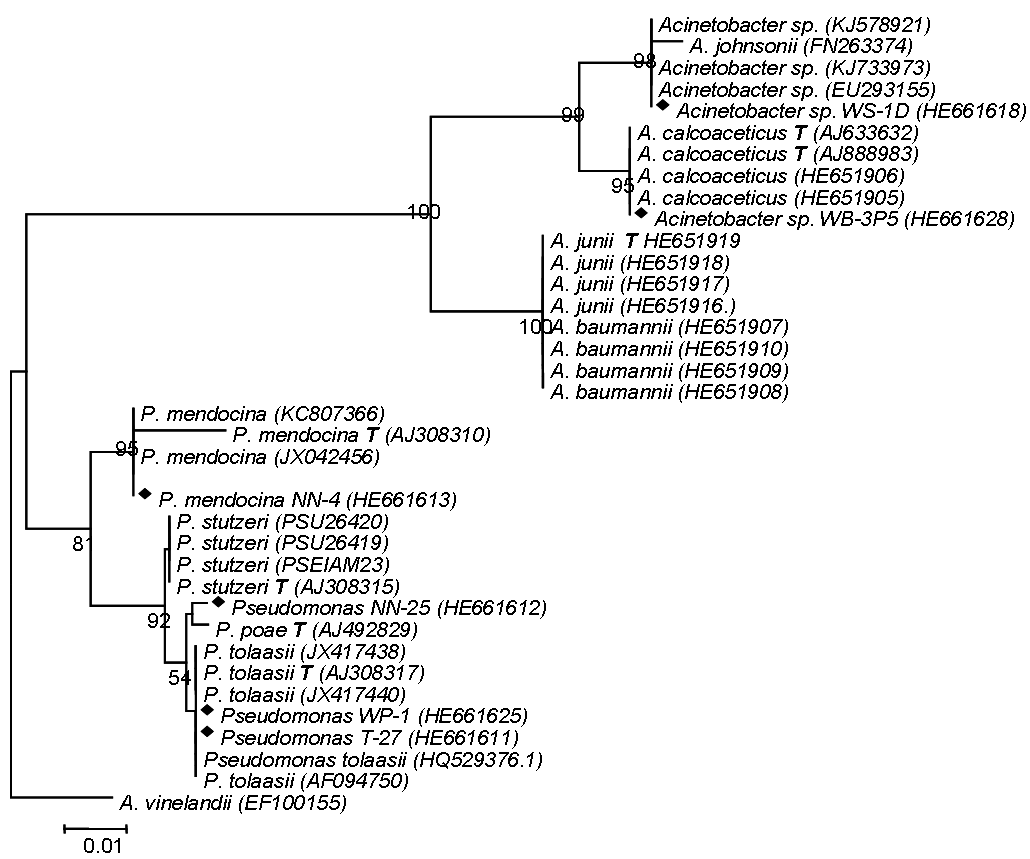

Supplement: S1 Fig — Amplified 16S rRNA gene fragments from the isolated strains of Acinetobacter and Pseudomonas were sequenced and BLAST searched through NCBI database. Closely related sequences were downloaded and aligned using CLUSTAL X. These sequences were analyzed using neighbor-joining method. The bootstrap replicates (BS) values of 50% or greater represent well supported nodes and thus only those were retained. Type strain of Azotobacter vinelandii was taken as outgroup. (TIF) [file pone.0130030.s001.tif]

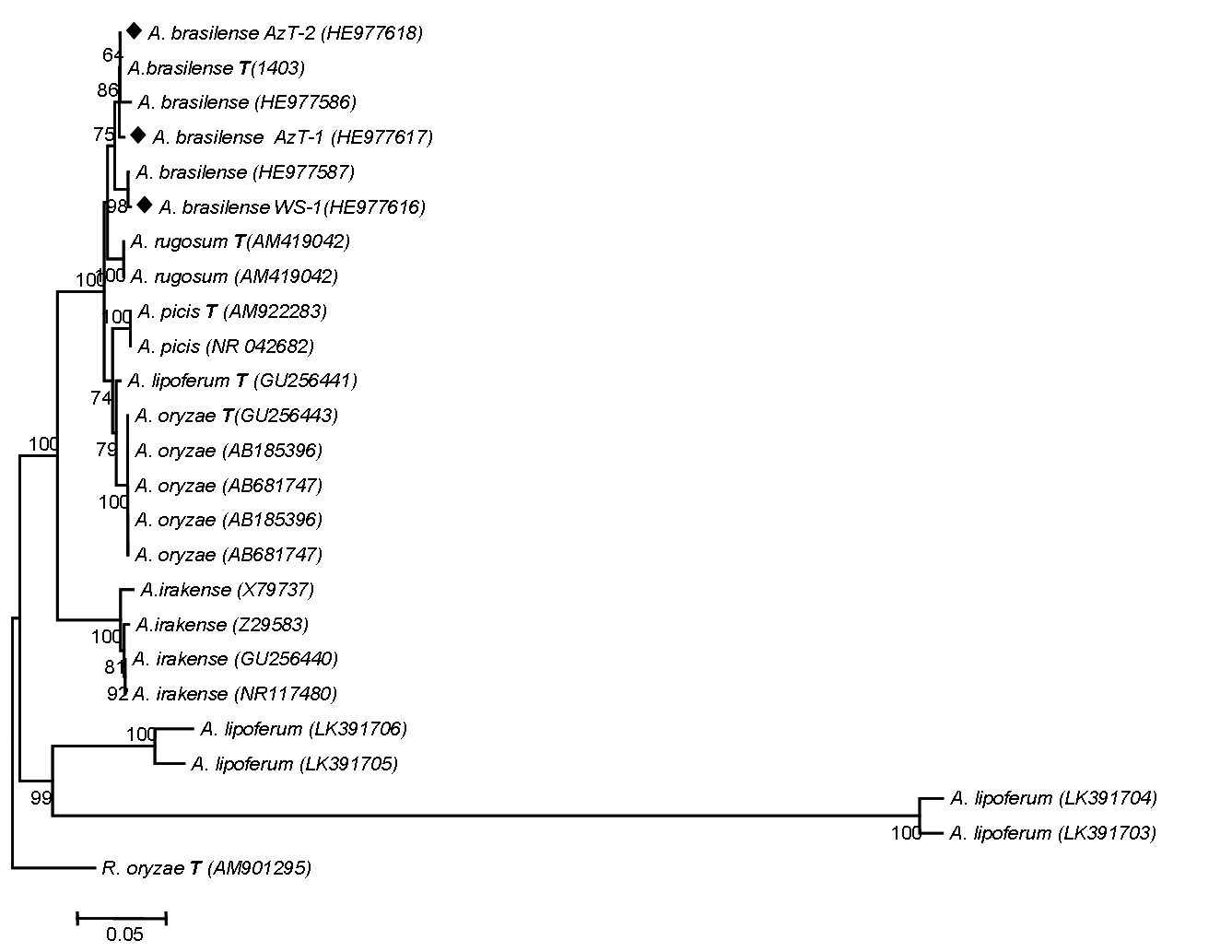

Supplement: S2 Fig — Amplified 16S rRNA gene fragments from the isolated strains of Azospirillum (WS-1, AzT-1 and AzT-2) were sequenced and BLAST searched through NCBI database. Closely related sequences were downloaded and aligned using CLUSTAL X. These sequences were analyzed using neighbor-joining method. The bootstrap replicates (BS) values of 50% or greater represent well supported nodes and thus only those were retained. Type strain of Rhodospirillum oryzae was taken as outgroup. (TIF) [file pone.0130030.s002.tif]

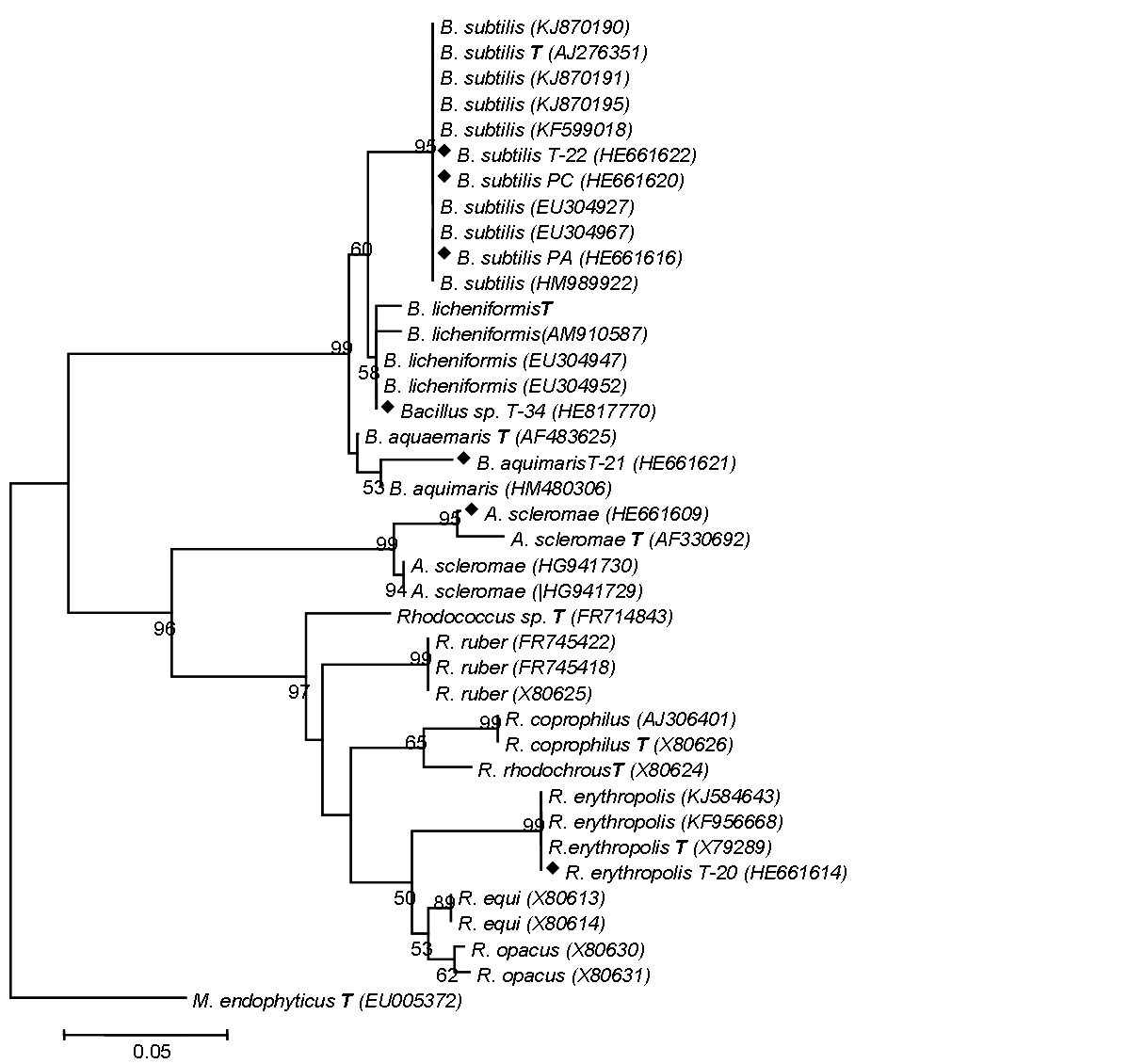

Supplement: S3 Fig — Amplified 16S rRNA gene fragments from the isolated strains PA, PC, T-21, T-22, T-34, WK2T and T-20 were sequenced and BLAST searched through NCBI database. Closely related sequences were downloaded and aligned using CLUSTAL X. These sequences were analyzed using neighbor-joining method. The bootstrap replicates (BS) values of 50% or greater represent well supported nodes and thus only those were retained. Type strain of Micrococcus endophyticus was taken as outgroup. (TIF) [file pone.0130030.s003.tif]

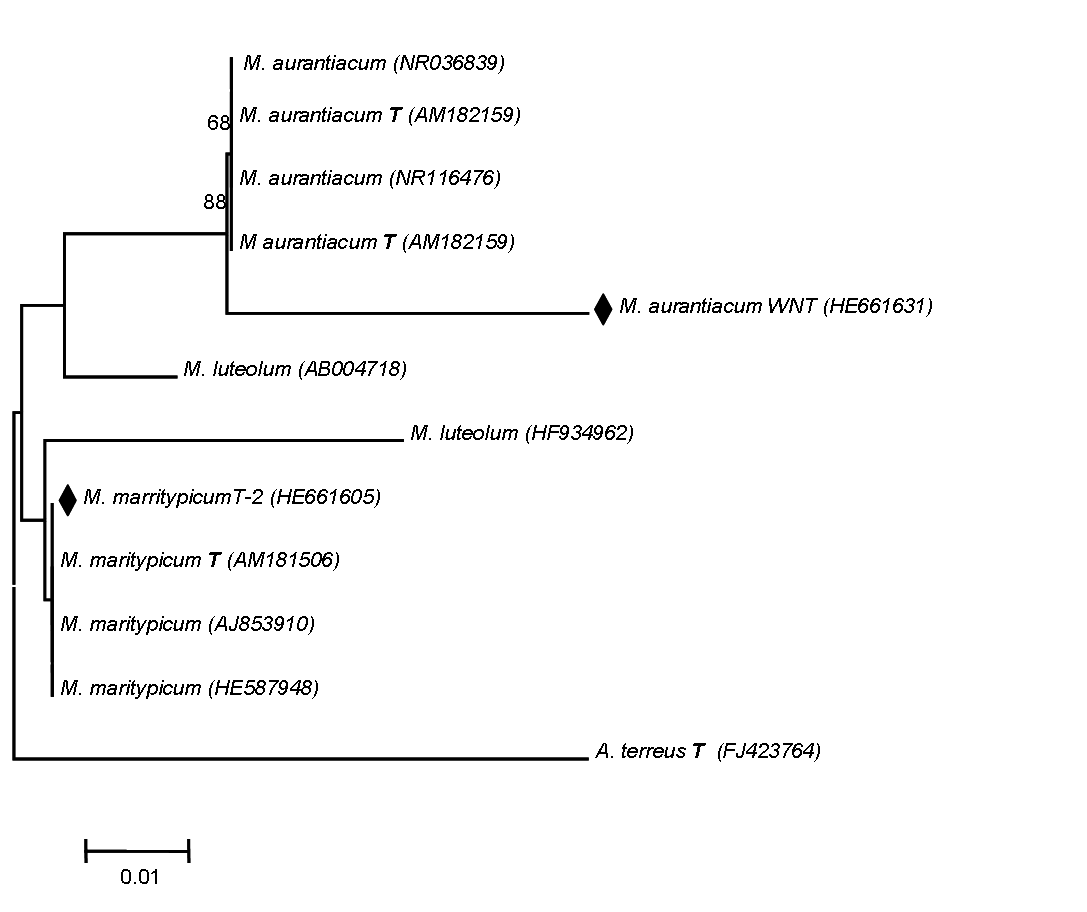

Supplement: S4 Fig — Amplified 16S rRNA gene fragments from the isolated strains WNT and T-2 were sequenced and BLAST searched through NCBI database. Closely related sequences were downloaded and aligned using CLUSTAL X. These sequences were analyzed using neighbor-joining method. The bootstrap replicates (BS) values of 50% or greater represent well supported nodes and thus only those were retained. Type strain of Agrococcus terreus was taken as outgroup. (TIF) [file pone.0130030.s004.tif]

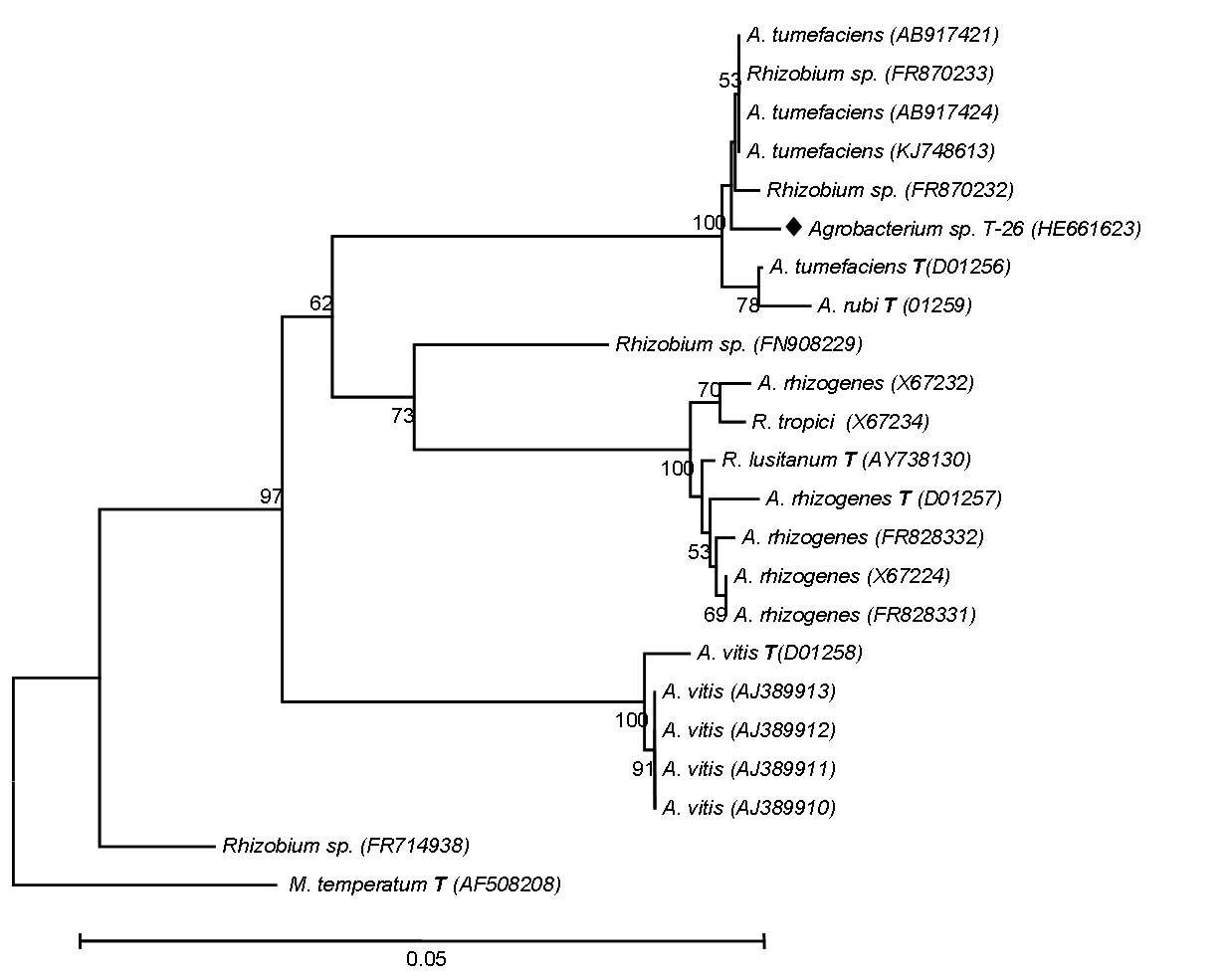

Supplement: S5 Fig — Amplified 16S rRNA gene fragments from the isolated strains T-26 was sequenced and BLAST searched through NCBI database. Closely related sequences were downloaded and aligned using CLUSTAL X. These sequences were analyzed using neighbor-joining method. The bootstrap replicates (BS) values of 50% or greater represent well supported nodes and thus only those were retained. Type strain of Mesorhizobium temperatum was taken as outgroup. (TIF) [file pone.0130030.s005.tif]

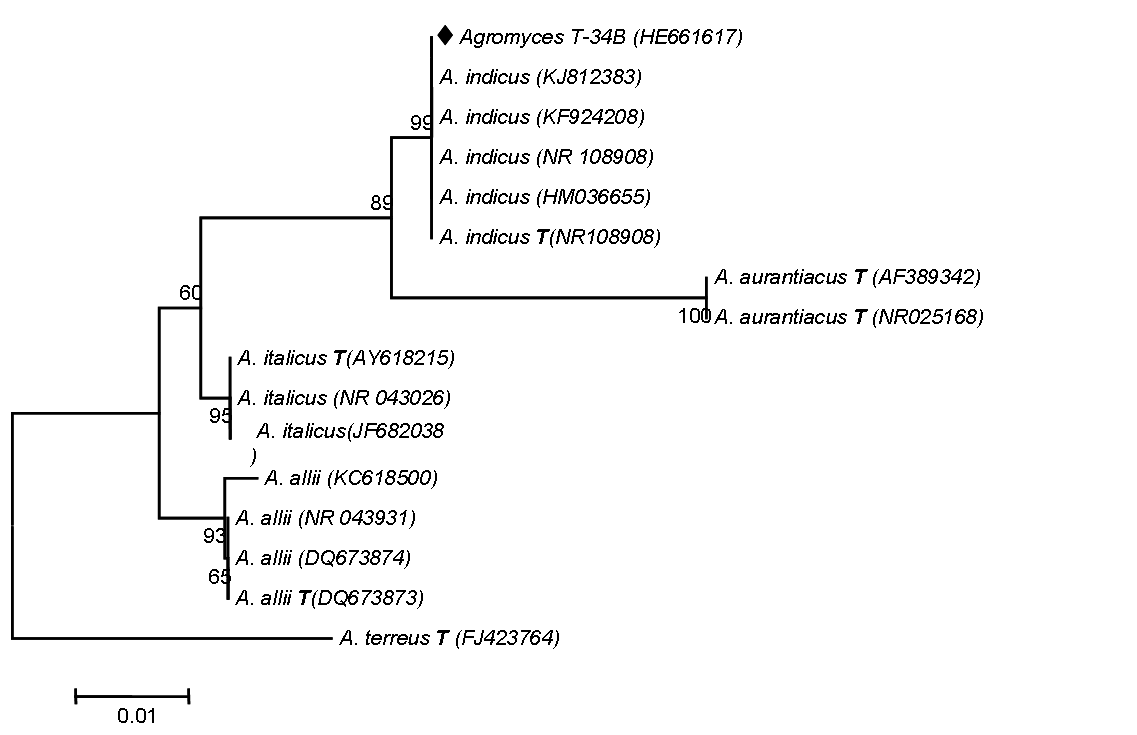

Supplement: S6 Fig — Amplified 16S rRNA gene fragments from the isolated strains T-34B was sequenced and BLAST searched through NCBI database. Closely related sequences were downloaded and aligned using CLUSTAL X. These sequences were analyzed using neighbor-joining method. The bootstrap replicates (BS) values of 50% or greater represent well supported nodes and thus only those were retained. Type strain of Agrococcus terreus was taken as outgroup. (TIF) [file pone.0130030.s006.tif]

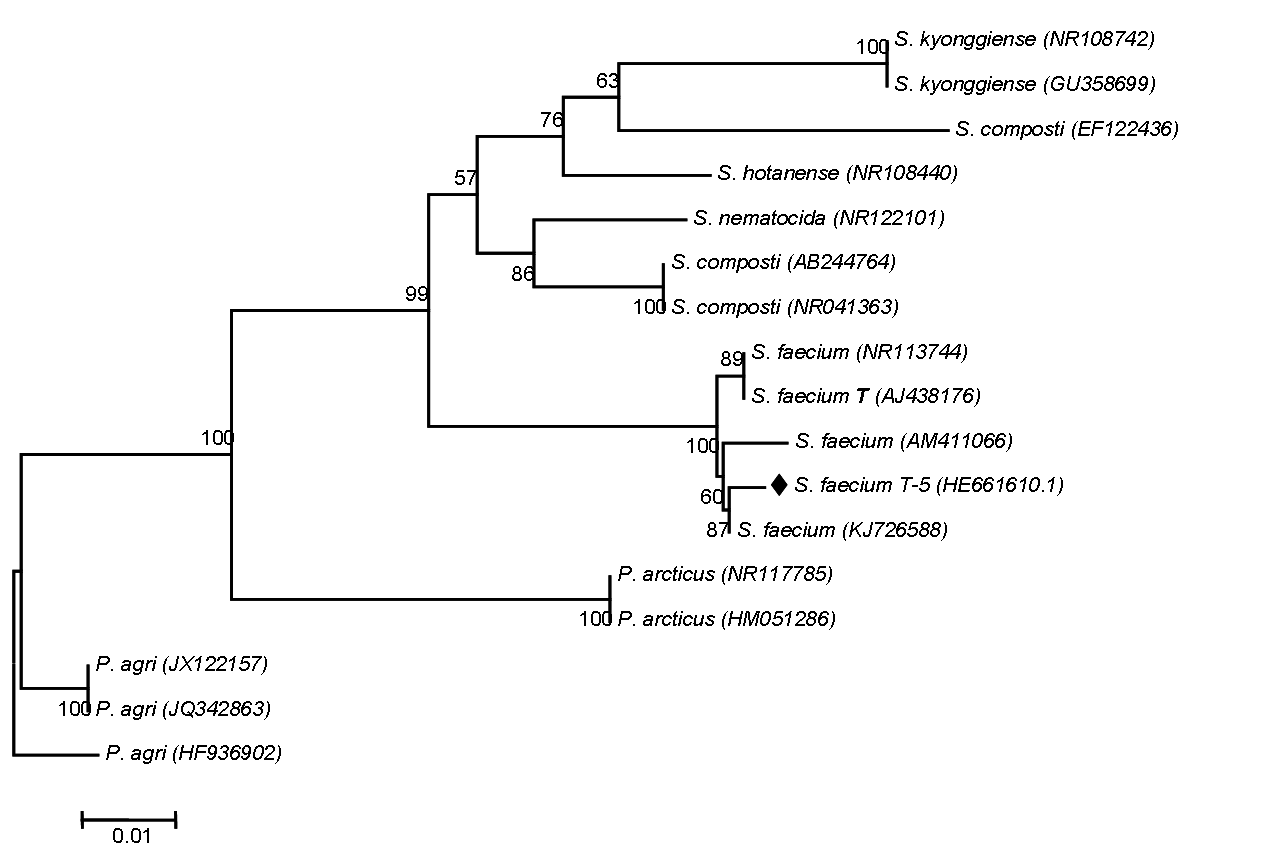

Supplement: S7 Fig — Amplified 16S rRNA gene fragments from the isolated strains T-5 was sequenced and BLAST searched through NCBI database. Closely related sequences were downloaded and aligned using CLUSTAL X. These sequences were analyzed using neighbor-joining method. The bootstrap replicates (BS) values of 50% or greater represent well supported nodes and thus only those were retained. Type strain of Pedobacter agri was taken as outgroup. (TIF) [file pone.0130030.s007.tif]

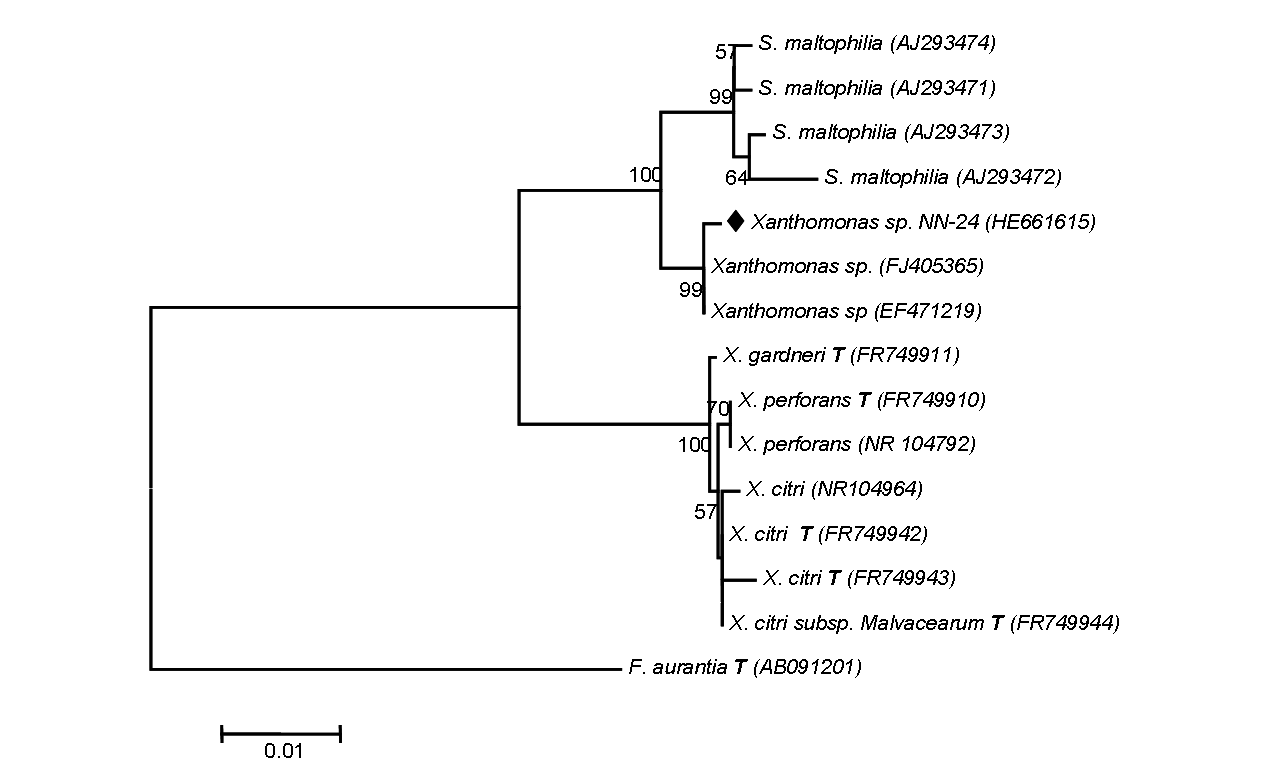

Supplement: S8 Fig — Amplified 16S rRNA gene fragments from the isolated strains NN-24 was sequenced and BLAST searched through NCBI database. Closely related sequences were downloaded and aligned using CLUSTAL X. These sequences were analyzed using neighbor-joining method. The bootstrap replicates (BS) values of 50% or greater represent well supported nodes and thus only those were retained. Type strain of Frateuria aurantia was taken as outgroup. (TIF) [file pone.0130030.s008.tif]

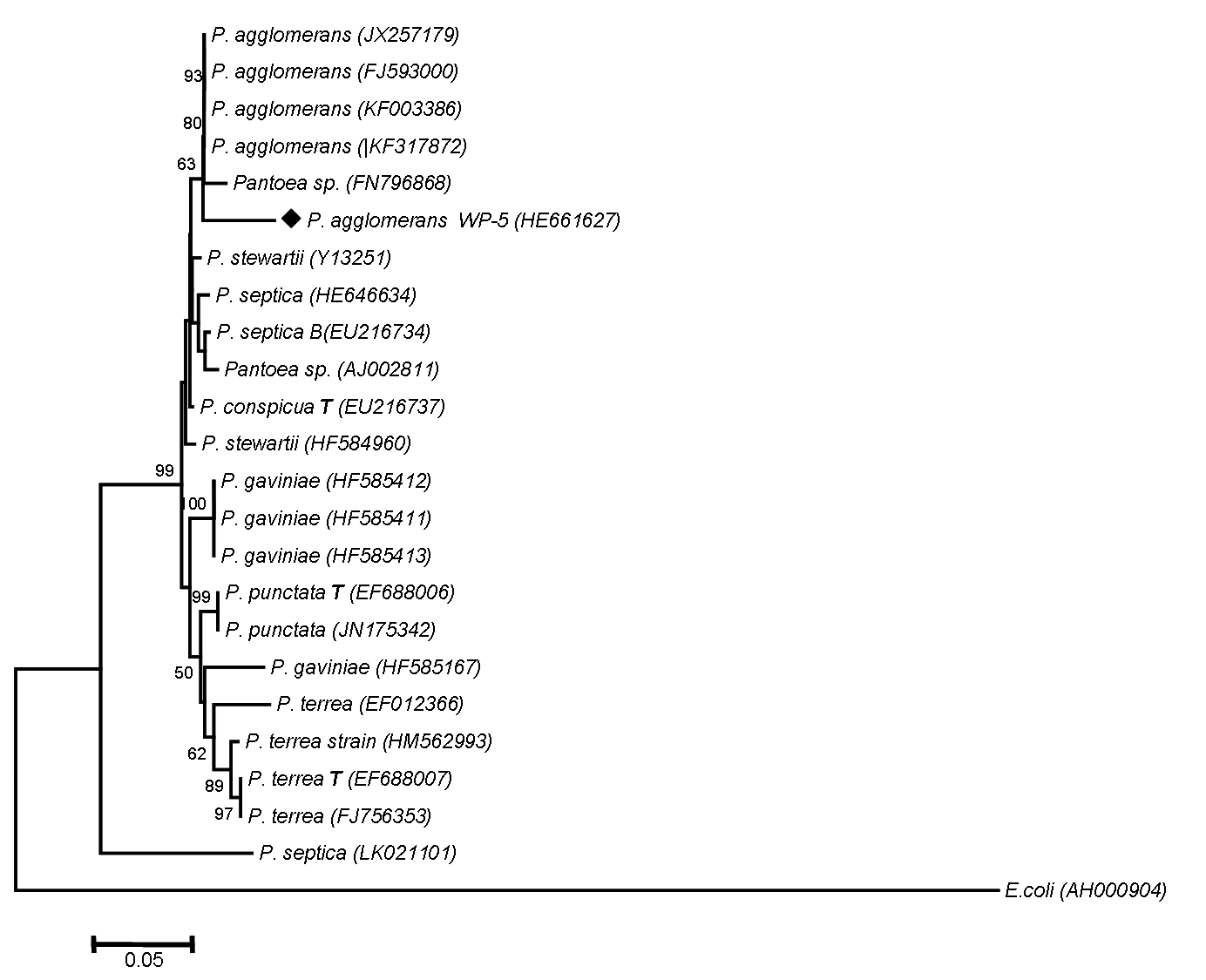

Supplement: S9 Fig — Amplified 16S rRNA gene fragments from the isolated strains ofWP-5 was sequenced and BLAST searched through NCBI database. Closely related sequences were downloaded and aligned using CLUSTAL X. These sequences were analyzed using neighbor-joining method. The bootstrap replicates (BS) values of 50% or greater represent well supported nodes and thus only those were retained. Type strain of E.coli was taken as outgroup. (TIF) [file pone.0130030.s009.tif]

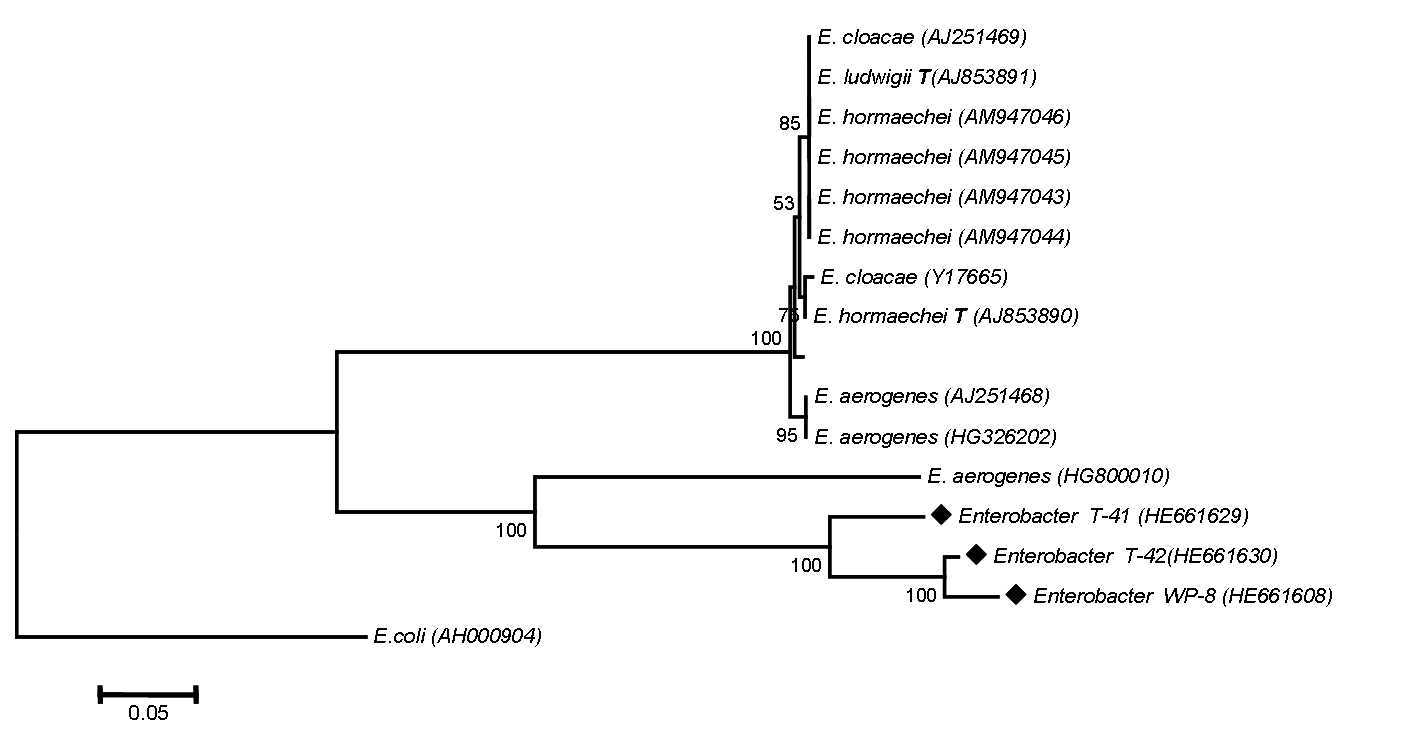

Supplement: S10 Fig — Amplified 16S rRNA gene fragments from the isolated strains ofT-41, T-42 and WP-8 was sequenced and BLAST searched through NCBI database. Closely related sequences were downloaded and aligned using CLUSTAL X. These sequences were analyzed using neighbor-joining method. The bootstrap replicates (BS) values of 50% or greater represent well supported nodes and thus only those were retained. Type strain of E.coli was taken as outgroup. (TIF) [file pone.0130030.s010.tif]
